# Identification and characterization of protein *N*-myristoylation occurring on four human mitochondrial proteins, SAMM50, TOMM40, MIC19, and MIC25

**DOI:** 10.1371/journal.pone.0206355

**Published:** 2018-11-14

**Authors:** Toshihiko Utsumi, Kanako Matsuzaki, Aya Kiwado, Ayane Tanikawa, Yuki Kikkawa, Takuro Hosokawa, Aoi Otsuka, Yoshihito Iuchi, Hirotsugu Kobuchi, Koko Moriya

**Affiliations:** 1 Graduate School of Sciences and Technology for Innovation, Yamaguchi University, Yamaguchi, Japan; 2 Department of Biological Chemistry, Faculty of Agriculture, Yamaguchi University, Yamaguchi, Japan; 3 Department of Cell Chemistry, Okayama University Graduate School of Medicine, Dentistry and Pharmaceutical Sciences, Okayama, Japan; University of Alabama at Birmingham, UNITED STATES

## Abstract

Previously, we showed that SAMM50, a mitochondrial outer membrane protein, is *N*-myristoylated, and this lipid modification is required for the proper targeting of SAMM50 to mitochondria. In this study, we characterized protein *N*-myristoylation occurring on four human mitochondrial proteins, SAMM50, TOMM40, MIC19, and MIC25, three of which are components of the mitochondrial intermembrane space bridging (MIB) complex, which plays a critical role in the structure and function of mitochondria. *In vitro* and *in vivo* metabolic labeling experiments revealed that all four of these proteins were *N*-myristoylated. Analysis of intracellular localization of wild-type and non-myristoylated G2A mutants of these proteins by immunofluorescence microscopic analysis and subcellular fractionation analysis indicated that protein *N*-myristoylation plays a critical role in mitochondrial targeting and membrane binding of two MIB components, SAMM50 and MIC19, but not those of TOMM40 and MIC25. Immunoprecipitation experiments using specific antibodies revealed that MIC19, but not MIC25, was a major *N*-myristoylated binding partner of SAMM50. Immunoprecipitation experiments using a stable transformant of MIC19 confirmed that protein *N*-myristoylation of MIC19 is required for the interaction between MIC19 and SAMM50, as reported previously. Thus, protein *N*-myristoylation occurring on two mitochondrial MIB components, SAMM50 and MIC19, plays a critical role in the mitochondrial targeting and protein-protein interaction between these two MIB components.

## Introduction

Protein *N*-myristoylation is one of the predominant forms of lipid modification that occurs on eukaryotic and viral proteins [1–6]. This modification is the attachment of myristic acid, a 14-carbon saturated fatty acid, to the N-terminal Gly of proteins. In general, myristic acid is cotranslationally attached to the N-terminal Gly residue after removal of the initiating Met. A stable amide bond links myristic acid irreversibly to the protein. In addition to cotranslational protein *N*-myristoylation, it is now established that posttranslational *N*-myristoylation can also occur on many caspase-cleavage products such as Bid, actin, gelsolin, and p21 activated kinases 2 (PAK2), in which proteolytic cleavage by caspase causes the exposure of an internal *N*-myristoylation motif [7–10]. Both cotranslational and posttranslational *N*-myristoylation is catalyzed by *N*-myristoyltransferase, a member of the GCN5-related *N*-acetyltransferase superfamily of proteins [11]. Many *N*-myristoylated proteins play critical roles in regulating cellular structure and function. These include proteins involved in a wide variety of cellular signal transduction pathways, such as guanine nucleotide binding proteins, protein kinases, phosphatases, E3-ubiquitin ligases, Ca^2+^ binding proteins, and cytoskeletal regulatory proteins. In addition to proteins related to cellular signal transduction pathways, recent studies have revealed that protein *N*-myristoylation occurs on many disease-associated proteins [12–16]. In many cases, the functions of these *N*-myristoylated proteins are regulated by reversible membrane binding mediated by protein *N*-myristoylation. Thus, protein *N*-myristoylation has been recognized as a protein modification that occurs mainly on cytoplasmic proteins, and only very few integral membrane proteins have been demonstrated to be *N*-myristoylated so far [17].

Previously, we showed that SAMM50, a mitochondrial outer membrane protein, is *N*-myristoylated, and this lipid modification is required for proper targeting of SAMM50 to mitochondria [18]. SAMM50 is an integral membrane protein with a β-barrel structure, and it is a central component of the sorting and assembly machinery (SAM) necessary for the assembly of β-barrel proteins in the mitochondrial outer membrane [19–21]. SAMM50 has been shown to interact with MIC19 (CHCHD3, MINOS3) and MIC25 (CHCHD6, CHCM1), *N*-myristoylated or putatively *N*-myristoylated mitochondrial intermembrane space protein, to form the mitochondrial intermembrane space bridging (MIB) complex that plays a critical role in the structure and function of mitochondria [22–25]. However, the relative amount of these *N*-myristoylated proteins present in the cell, or the role of protein *N*-myristoylation in the function of these proteins has not been fully elucidated. These two proteins are components of mitochondrial contact site and cristae organizing system (MICOS) complex that plays critical role in cristae formation of the inner mitochondrial membrane and communication with the outer mitochondrial membrane [23, 24, 26]. MICOS complex interacts with components of the SAM complex of the outer membrane to form MIB complex [22–26]. In this study, we examined protein *N*-myristoylation occurring on four human mitochondrial proteins, SAMM50, TOMM40, MIC19, and MIC25. Among them, SAMM50, MIC19, and MIC25 are MIB components and TOMM40 is a major component of the translocase of the mitochondrial outer membrane (TOM complex) acts as a general import pore for most mitochondrial precursor proteins [20, 27].

As a result, it was revealed that all of these four proteins are *N*-myristoylated proteins. Analysis of intracellular localization indicated that protein *N*-myristoylation plays a critical role in mitochondrial targeting and membrane binding of two MIB components, SAMM50 and MIC19, but not those of TOMM40 and MIC25. Immunoprecipitation experiments using specific antibodies revealed MIC19, but not MIC25, to be a major *N*-myristoylated binding partner of SAMM50. Several molecules of MIC19 seemed to bind to each SAMM50 molecule. It was also revealed that 25 kDa MIC19 and its ~32 kDa isoform were ubiquitously expressed in mammalian cells. However, the expression of MIC25 differs depending on the cell type, and only very low level of expression was detected in human HeLa cells. Immunoprecipitation experiments using a stable transformant of MIC19 revealed that protein *N*-myristoylation of MIC19 plays a critical role in the interaction between MIC19 and SAMM50, as reported previously [24]. Thus, protein *N*-myristoylation occurring on two mitochondrial MIB components, SAMM50 and MIC19, plays a critical role in mitochondrial targeting and protein-protein interaction between these two MIB components.

## Materials and methods

### Materials

Human cDNAs (Flexi ORF clones) were purchased from Promega (Madison, WI, USA). Transdirect insect cell, an insect cell-free protein synthesis system, was obtained from Shimadzu (Kyoto, Japan). Trident Membrane Protein Extraction Kit was from GeneTex (Irvine, CA, USA). A T7-Scribe standard RNA IVT kit was from CELLSCRIPT (Madison, WI, USA). [^3^H]leucine, [^3^H]myristic acid, and ECL prime Western blotting detection reagent were from GE Healthcare (Buckinghamshire, UK). ImmunoStar LD Western blotting detection reagent was from Wako Pure Chemical (Osaka, Japan). ENLIGHTENING was from PerkinElmer (Waltham, MA, USA). The dye terminator cycle sequencing kit, Lipofectamine LTX and Plus reagents, MitoTracker Red CMXRos, and Hoechst 33342 were from Life Technologies Corporation (Carlsbad, CA, USA). Anti-FLAG monoclonal antibody, anti-SAMM50 monoclonal antibody (WH0025813), anti-SAMM50 polyclonal antibody (HPA034537), anti-TOMM40 polyclonal antibody (HPA036231), anti-MIC19 polyclonal antibody (HPA042835), anti-MIC25 polyclonal antibody (HPA047673), and anti-rabbit IgG-FITC antibody were from Sigma (St. Louis, MO, USA). 13-tetradecynoic acid (Alk-Myr) was from Cayman (Ann Arbor, MI, USA). Azide TAMRA (Az-TAMRA) was from Click Chemistry Tools (Scottsdale, AZ, USA). Tris(2-carboxyethyl)phosphine hydrochloride (TCEP) and tris[(1-benzyl-1*H*-1,2,3-triazol-4-yl)methyl]amine (TBTA) were from Sigma (St. Louis, MO, USA). Protein G-HRP conjugate was from Bio-Rad (Hercules, CA, USA). X-ray film was from Eastman Kodak (Rochester, NY, USA). The other reagents used were from Wako Pure Chemical (Osaka, Japan), Daiichi Pure Chemicals (Tokyo, Japan) or Seikagaku Kogyo (Tokyo, Japan) and were of analytical or DNA grade.

### Prediction of protein *N*-myristoylation using prediction programs

Two public WWW-server-based prediction programs for protein *N*-myristoylation, MYR Predictor (http://mendel.imp.ac.at/myristate/SUPLpredictor.htm) [28] and Myristoylator (http://www.expasy.org/tools/myristoylator/) [29], were used for the prediction of protein *N*-myristoylation. The entire amino acid sequences deduced from the nucleotide sequences of the ORFs were used as the query.

### Plasmid construction

Nucleotide sequences of oligonucleotide primers used for plasmid construction are summarized in S1 Table. Plasmid pTD1 (Shimadzu) was used for the insect cell-free protein synthesis system [30]. Plasmids pTD1-SAMM50-, SAMM50-G2A-FLAG were constructed as described previously [18]. Construction of the pTD1 plasmids including cDNAs encoding FLAG-tagged proteins of TOMM40 and TOMM40-G2A are summarized in S2 Table. Construction of the pcDNA3 plasmids including cDNAs encoding FLAG-tagged or tag-free proteins of SAMM50, TOMM40, MIC19, MIC25 and their G2A mutants are summarized in S2 Table.

### *In vitro* transcription reaction

mRNAs encoding the cDNAs were prepared using a T7-scribe standard RNA IVT kit (CELLSCRIPT) in accordance with the manufacturer’s instructions. The synthesized mRNAs were purified by phenol-chloroform extraction and ethanol precipitation before use in the translation reaction.

### Cell-free protein synthesis

The translation reaction was performed using an insect cell-free protein synthesis system (Shimadzu) in the presence of [^3^H]leucine or [^3^H]myristic acid as described previously [31, 32]. The mixture (composed of 6.2 μL insect cell lysate, 3.7 μL reaction buffer, 0.5 μL 4 mM methionine, 1.0 μL [^3^H]leucine [1 μCi] or 3.0 μL [^3^H]myristic acid [20 μCi], and 2 μL mRNA [8 μg]) was incubated at 26°C for 6 h. The translation products were then analyzed by SDS-PAGE and fluorography.

### Transfection of cells

COS-1 (simian virus 40-transformed African green monkey kidney cell line, American Type Culture Collection) cells, HEK293T (a human embryonic kidney cell line) cells, HeLa cells, or HepG2 cells were maintained in Dulbecco’s modified Eagle’s medium (DMEM; Gibco BRL [Palo Alto, CA, USA]) supplemented with 10% fetal calf serum (FCS; Gibco BRL). Cells (2 × 10^5^) were plated onto 35-mm diameter dishes 1 day before transfection. pcDNA3 constructs (2 μg) containing cDNAs encoding FLAG-tagged or tag-free proteins were used to transfect the cells in each plate along with 2.5 μL Lipofectamine LTX and 2 μL Plus reagent in 1 mL serum-free medium [33]. After incubation for 5 h at 37°C, the cells were re-fed with serum-containing medium and incubated again at 37°C for appropriate periods. Selection of cells stably expressing transfected cDNA was performed as described previously [34].

### Metabolic labeling of cells

The metabolic labeling of cells with [^3^H]myristic acid or myristic acid analog (Alk-Myr) was performed as described previously [32, 35]. Cells (2 × 10^5^) were transfected with pcDNA3 constructs (2 μg) containing cDNAs as described above, and incubated at 37°C for 12 h. Then, they were washed once with 1 mL serum-free DMEM and incubated for 10 h at 37°C in 1 mL DMEM (+2% FCS) containing [^3^H]myristic acid (100 μCi/mL) or 25 μM Alk-Myr. Subsequently, the cells were washed three times with Dulbecco’s phosphate-buffered saline (DPBS), harvested and lysed with 200 μL of RIPA buffer (50 mM Tris-HCl (pH 7.5), 150 mM NaCl, 1% Nonidet P-40, 0.5% sodium deoxycholate, 0.1% SDS, protease inhibitors) on ice for 20 min. The radiolabeled samples were then analyzed by SDS-PAGE and fluorography. The samples labeled with Alk-Myr were reacted with Az-TAMRA by click chemistry, and then protein *N*-myristoylation was analyzed by in-gel fluorescence analysis.

### SDS-PAGE and fluorography

The radiolabeled proteins were resolved by 12.5% SDS-PAGE, then the gel was fixed and soaked in ENLIGHTENING (PerkinElmer) for 20 min. Thereafter, the gel was dried under vacuum and exposed to X-ray film for an appropriate period.

### Cu(I)-catalyzed azide-alkyne cycloaddition (CuAAC)

The cell lysates labeled with Alk-Myr (46 μL) were reacted with 4 μL of freshly premixed click chemistry reaction cocktail (1 μL Az-TAMRA [5 mM], 1 μL TCEP [50 mM], 1 μL TBTA [5 mM], and 1 μL CuSO_4_•5H_2_O [50 mM]) in a total reaction volume of 50 μL for 1 h at room temperature. After CuAAC, 500 μL of MeOH was added to the sample, then the samples were placed at -80°C overnight. After centrifugation at 15,000 rpm at 4°C for 30 min, the supernatant was removed. Thereafter, the pellet was washed with 500 μL of MeOH, and then dried in air. The samples were denatured by sonication in SDS-sample buffer, and then subjected to SDS-PAGE. In-gel fluorescence analysis of the gel obtained by SDS-PAGE was performed using a Typhoon FLA9500 (GE-Healthcare Bio-Sciences AB, Uppsala, Sweden).

### Western blotting

Proteins were resolved by 12.5% SDS-PAGE and then transferred to an Immobilon-P transfer membrane. After blocking with non-fat milk, the membrane was probed with a primary antibody, as described previously [36]. Immunoreactive proteins were detected specifically by incubation with protein G-HRP conjugate. The membrane was developed using ECL Prime or ImmunoStar LD western blotting detection reagent and detected using a MicroChemi Chemiluminescence Imaging System (Berthold Technologies, Bad Wildbad, Germany). The blots were quantified by densitometry using the software TotalLab Quant (TotalLab Limited, Newcastle upon Tyne, UK).

### Isolation of total membrane proteins and cytosolic proteins in cells

Isolation of total membrane proteins and cytosolic proteins in intact or transfected COS-1 cells was performed using Trident Membrane Protein Extraction Kit (Gene Tex) according to the manufacturer’s protocol. The obtained total membrane protein fraction and cytosolic fraction were subjected to western blotting analysis.

### Immunofluorescence analysis and fluorescence microscopy

Immunofluorescence analysis of transfected cells was performed 24 h after transfection [37]. After staining with Hoechst 33342 and MitoTracker Red, the cells were washed with DPBS, fixed in 4% paraformaldehyde in DPBS for 15 min, and permeabilized with 0.1% Triton X-100 in DPBS for 10 min at room temperature, followed by washing with 0.1% gelatin in DPBS. The permeabilized cells were incubated with a specific antibody in DPBS for 1 h at room temperature. After washing with 0.1% gelatin in DPBS, the cells were incubated with anti-rabbit IgG-FITC antibody for 1 h at room temperature. After washing with 0.1% gelatin in DPBS, the cells were observed using a Leica AF7000 fluorescence microscope (Leica, Solmser, Germany).

### Immunoprecipitation

Samples were immunoprecipitated with specific antibodies as described previously [18].

### Statistical analysis

Statistical analysis was carried out using two-tailed *t* test (Microsoft Excel; Microsoft). The means of two distributions were considered significantly different if p < 0.05.

## Results

### Identification of protein *N*-myristoylation occurring on human SAMM50 and TOMM40 by cell-free and cellular metabolic labeling

**Fig 1:**
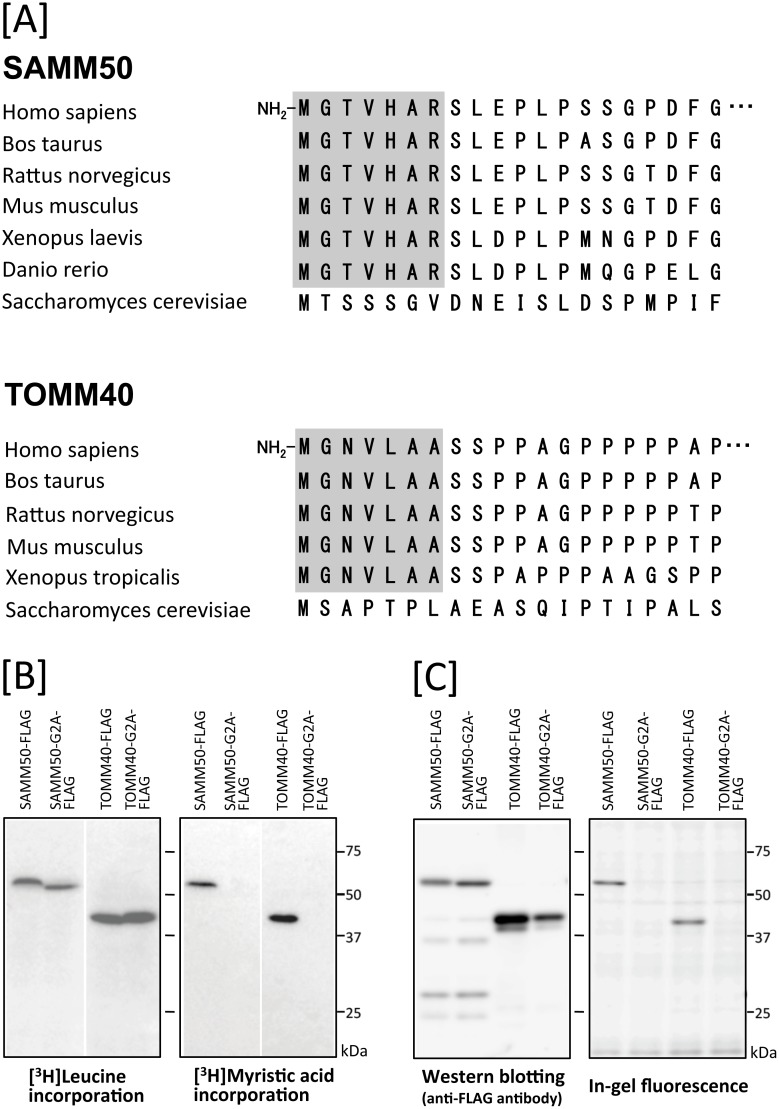
Detection of protein *N*-myristoylation of human SAMM50 and TOMM40 by cell-free and cellular metabolic labeling. A. Interspecies alignment of the N-terminal sequences of SAMM50 and TOMM40. *N*-myristoylation motifs are shown in grey in the N-terminal sequences. B. The gene products of cDNAs encoding SAMM50-FLAG, SAMM50-G2A-FLAG, TOMM40-FLAG, and TOMM40-G2A-FLAG were synthesized using an insect cell-free protein synthesis system in the presence of [^3^H]leucine or [^3^H]myristic acid. The labeled translation products were analyzed by SDS-PAGE and fluorography, as described in the Materials and methods. C. cDNAs encoding SAMM50-FLAG, SAMM50-G2A-FLAG, TOMM40-FLAG, and TOMM40-G2A-FLAG were transfected into COS-1 cells. The expression of proteins was evaluated by western blotting analysis using an anti-FLAG antibody. Protein *N*-myristoylation was evaluated by metabolic labeling with a myristic acid analog followed by click chemistry, as described in the Materials and methods.

We showed previously that human SAMM50, a mitochondrial β-barrel membrane protein, is *N*-myristoylated, and protein *N*-myristoylation is required for the proper targeting of SAMM50 to mitochondria [18]. When the N-terminal sequence of other human mitochondrial β-barrel membrane proteins was investigated, it was found that TOMM40, a major component of the translocase of the mitochondrial outer membrane (TOM complex), contained *N*-myristoylation motif at its N-terminus. In fact, both of two well-known *N*-myristoylation prediction programs (The MYR Predictor and Myristoylator) predicted TOMM40 to be an *N*-myristoylated protein. In addition, interspecies alignments of the N-terminal sequence of proteins revealed that the N-terminal *N*-myristoylation motif of TOMM40 is highly conserved among vertebrates, as is the case with SAMM50 (Fig 1A). To determine whether TOMM40 is *N*-myristoylated, cell-free and cellular metabolic labeling experiments were performed using cDNA coding for C-terminally FLAG-tagged TOMM40. For this analysis, non-*N*-myristoylated G2A mutant was constructed, in which Gly2 was replaced with Ala, and its susceptibility to protein *N*-myristoylation was compared with that of wild-type protein. As shown in Fig 1B, left panel, efficient protein expression ([^3^H]leucine incorporation) in the insect cell-free protein synthesis system was observed for the wild-type and G2A mutant forms of TOMM40-FLAG, as was the case with those of SAMM50-FLAG. Metabolic labeling with [^3^H]myristic acid revealed that efficient incorporation of [^3^H]myristic acid was observed in wild-type TOMM40-FLAG and SAMM50-FLAG, but incorporation was completely inhibited by replacing Gly2 with Ala (Fig 1B, right panel). In the case of cellular metabolic labeling experiments in COS-1 cells using a bioorthogonal myristic acid analog (Alk-Myr) followed by detection with click chemistry, results were obtained similar to those from the cell-free protein synthesis system, as shown in Fig 1C. These results clearly showed that TOMM40 and SAMM50 are *N*-myristoylated in *in vitro* and *in vivo* expression systems.

### Protein *N*-myristoylation of SAMM50 but not of TOMM40 is required for proper targeting to mitochondria

In order to determine the physiological role of protein *N*-myristoylation occurring on human SAMM50 and TOMM40, the role of protein *N*-myristoylation in the intracellular localization of these two proteins was investigated by immunofluorescence staining of COS-1 (simian virus 40-transformed African green monkey kidney cell line) cells transfected with these cDNAs. Both of SAMM50 and TOMM40 are β-barrel proteins and contain a β-signal near the C-terminus that is required for β-barrel precursor recognition by the SAM complex [38]. Thus, it is probable that the epitope-tagging at the C-terminus might affect the mitochondrial targeting of these proteins. Therefore, we used cDNAs encoding tag-free SAMM50 and TOMM40 to transfect COS-1 cells to investigate the intracellular localization of these two proteins.

**Fig 2:**
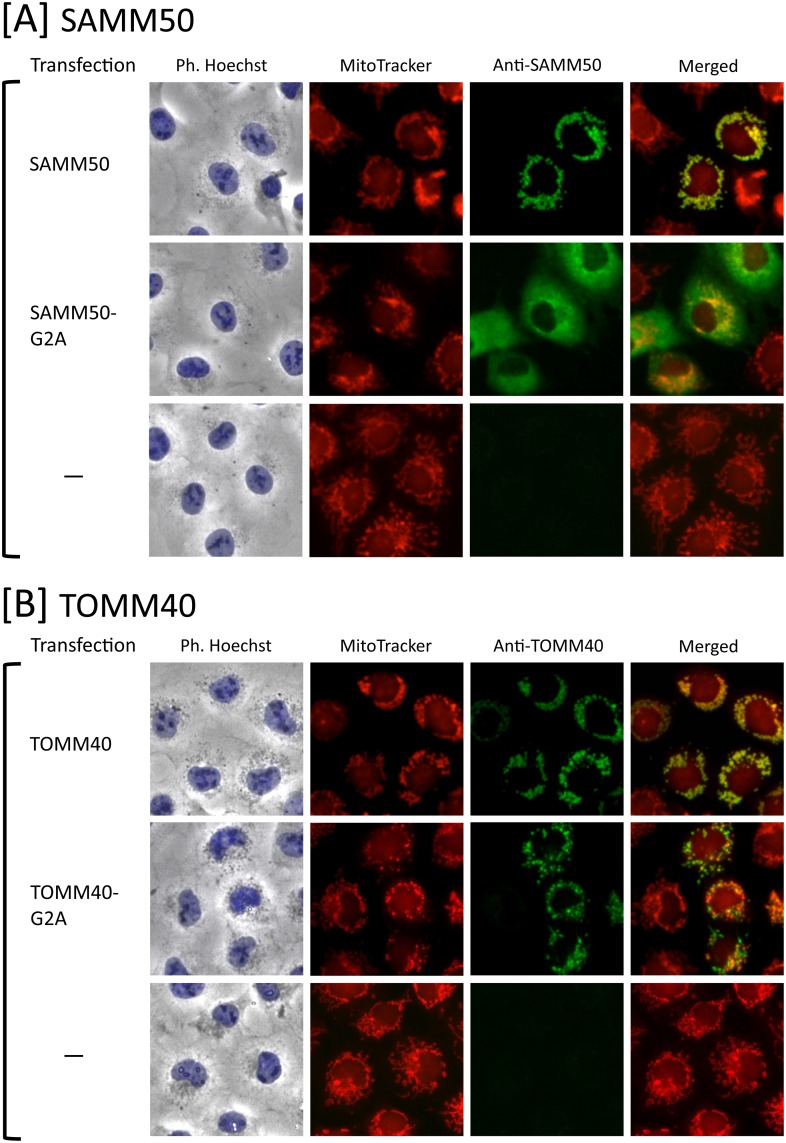
Protein *N*-myristoylation of SAMM50 but not of TOMM40 is required for proper targeting to mitochondria. A. Intracellular localization of SAMM50 and SAMM50-G2A was determined by immunofluorescence staining of COS-1 cells transfected with cDNAs encoding these two proteins using an anti-SAMM50 antibody. Mitochondria were detected using MitoTracker Red. The same experiment was performed using non-transfected COS-1 cells as a control. B. Intracellular localization of TOMM40 and TOMM40-G2A was determined by immunofluorescence staining of COS-1 cells transfected with cDNAs encoding these two proteins using an anti-TOMM40 antibody. Mitochondria were detected using MitoTracker Red. The same experiment was performed using non-transfected COS-1 cells as a control.

As shown in Fig 2A and 2B, a striking difference in the role of protein *N*-myristoylation in mitochondrial targeting was observed between SAMM50 and TOMM40. Immunofluorescence staining with the anti-SAMM50 antibody coupled with MitoTracker staining revealed that *N*-myristoylated SAMM50 exclusively localized to the mitochondria, whereas the non-myristoylated G2A mutant localized mainly to the cytoplasm. In contrast, in the case of TOMM40, both the wild-type and G2A mutant were found to localize exclusively to mitochondria. Thus, *N*-myristoylation of TOMM40 did not play a critical role in the mitochondrial targeting of TOMM40. In these experiments, endogenous SAMM50 and TOMM40 in control non-transfected COS-1 cells were not detected under the same experimental condition used for the analysis of the transfected COS-1 cells as shown in the lower panels of Fig 2A and 2B.

**Fig 3:**
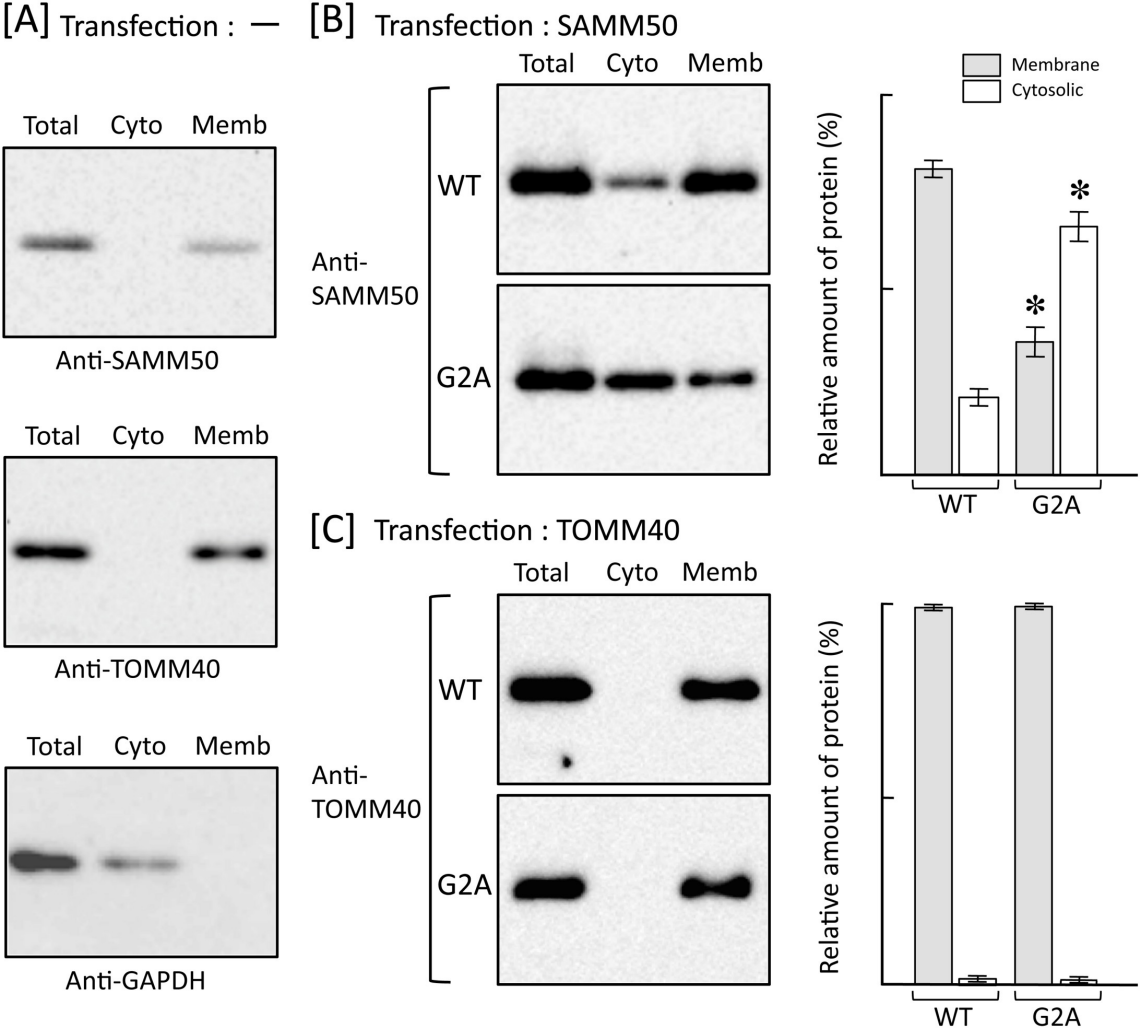
Protein *N*-myristoylation of SAMM50 but not of TOMM40 is required for binding of protein to the membrane. Total cell extracts of non-transfected COS-1 cells [A] and COS-1 cells transfected with cDNA encoding tag-free SAMM50 [B] or TOMM40 [C] were separated into cytosolic and membrane fractions by using membrane protein extraction kit (Trident). Presence of SAMM50 and TOMM40 in each fraction was determined by western blotting analysis using anti-SAMM50 or anti-TOMM40 antibody, respectively. For this analysis, anti-GAPDH antibody was used to detect GAPDH, a cytoslic marker protein [A]. For the analysis of COS-1 cells transfected with cDNAs coding for SAMM50 [B] and TOMM40 [C], relative amount of protein (%) reside in the cytosolic and total membrane fraction was calculated from densitometric analysis as described in materials and methods. Data are expressed as mean ± SD for three independent experiments. *P < 0.005 vs. wild-type.

In order to further clarify the role of protein *N*-myristoylation in the intracellular localization of SAMM50 and TOMM40, the role of protein *N*-myristoylation in the membrane binding of these two proteins was investigated by subcellular fractionation analysis. For this analysis, membrane protein extraction kit (Trident) was used to separate the total cell extracts into cytosolic and total membrane fractions. As shown in Fig 3A, endogenous SAMM50 and TOMM40 expressed in control non-transfected COS-1 cells were detected exclusively in total membrane fraction, whereas GAPDH, a cytosolic marker protein, was detected exclusively in cytosolic fraction. These data clearly indicated that the membrane proteins were successfully separated from the cytosolic proteins by using membrane protein extraction kit. As shown in Fig 3B and 3C, a striking difference in the role of protein *N*-myristoylation in membrane binding was observed between SAMM50 and TOMM40. Western blotting analysis using anti-SAMM50 antibody revealed that most of the overexpressed *N*-myristoylated SAMM50 localized to the membrane, whereas more than half of the non-myristoylated G2A mutant localized to the cytoplasm. In contrast, as for TOMM40, both the wild-type and G2A mutant were found to localize exclusively to the membrane. Thus, *N*-myristoylation of SAMM50 but not of TOMM40 plays positive role in the binding of protein to the membrane.

### Endogenous SAMM50 and TOMM40 expressed in mammalian cells is *N*-myristoylated

**Fig 4:**
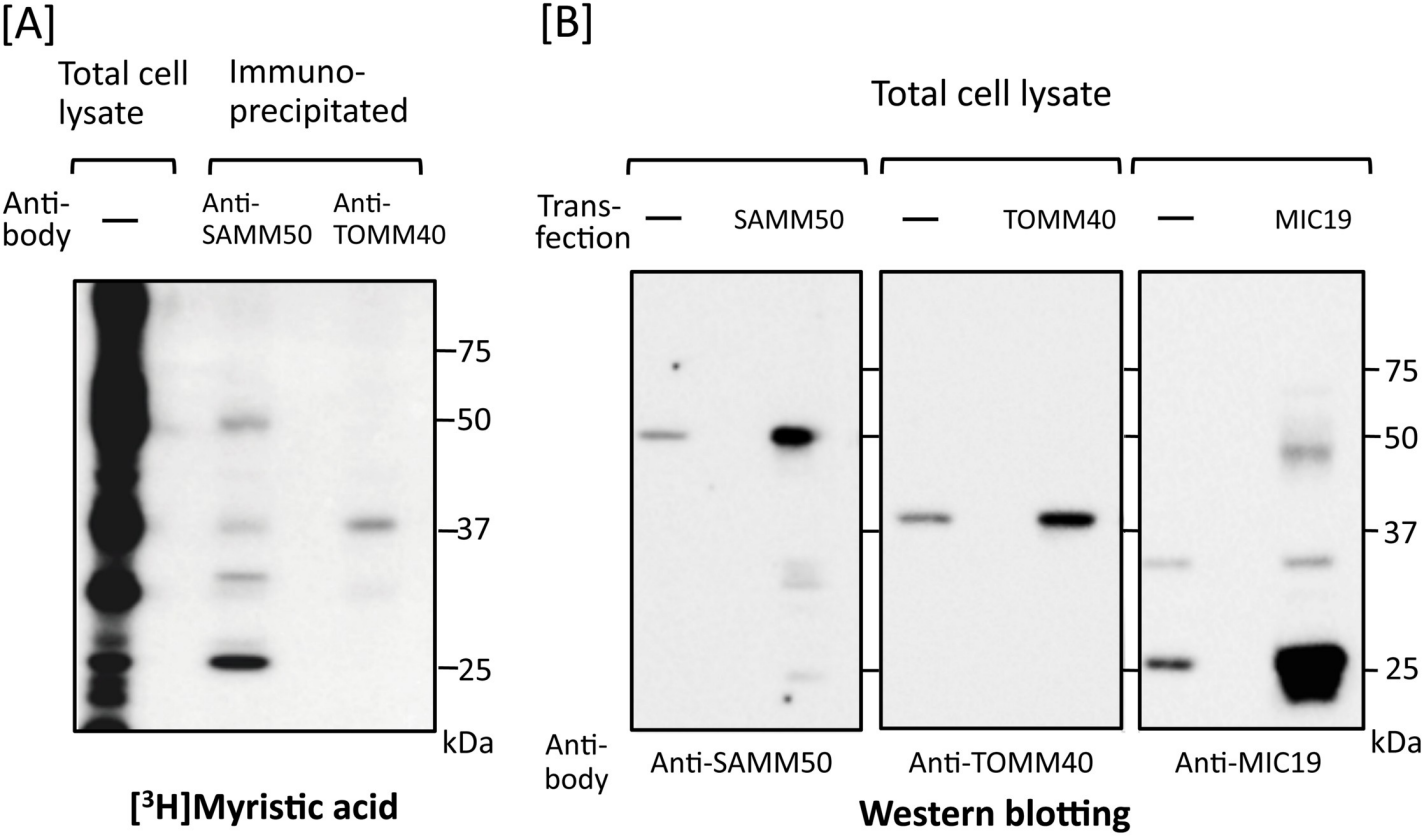
Endogenous SAMM50 and TOMM40 expressed in mammalian cells are *N*-myristoylated. A. Metabolic labeling of endogenous proteins expressed in COS-1 cells with [^3^H]myristic acid followed by immunoprecipitation with specific antibodies against SAMM50 and TOMM40 was performed. The labeled proteins were separated by SDS-PAGE and then detected by fluorography. B. Expression of endogenous SAMM50, TOMM40, and MIC19 in COS-1 cells was determined by western blotting analysis using the respective specific antibodies. For this analysis, tag-free-SAMM50, tag-free-TOMM40, and tag-free-MIC19 exogenously expressed in COS-1 cells were used as controls.

To determine whether protein *N*-myristoylation was observed on endogenous SAMM50 and TOMM40 expressed in mammalian cells, metabolic labeling of endogenous proteins expressed in COS-1 cells with [^3^H]myristic acid followed by immunoprecipitation with a specific antibody against these two proteins was performed. As shown in Fig 4A, lane 2 and 3, [^3^H]myristic acid-labeled protein bands with the expected molecular masses of SAMM50 (50 kDa) and TOMM40 (40 kDa) were detected by SDS-PAGE and fluorography. When western blotting analysis of total cell lysates of COS-1 cells were performed, a specific protein bands with molecular masses of 50 kDa and 40 kDa were detected using anti-SAMM50 and anti-TOMM40 antibodies, respectively (Fig 4B, lanes 1 and 3). In addition, the molecular masses of endogenous SAMM50 and TOMM40 were exactly the same as those observed with tag-free SAMM50 and TOMM40 overexpressed in COS-1 cells (Fig 4B, lanes 2 and 4), suggesting that the [^3^H]myristic acid-labeled protein bands with molecular masses of 50 kDa and 40 kDa were endogenous *N*-myristoylated SAMM50 and TOMM40, respectively.

### Identification of *N*-myristoylated proteins specifically interacting with *N*-myristoylated SAMM50 expressed in mammalian cells

**Fig 5:**
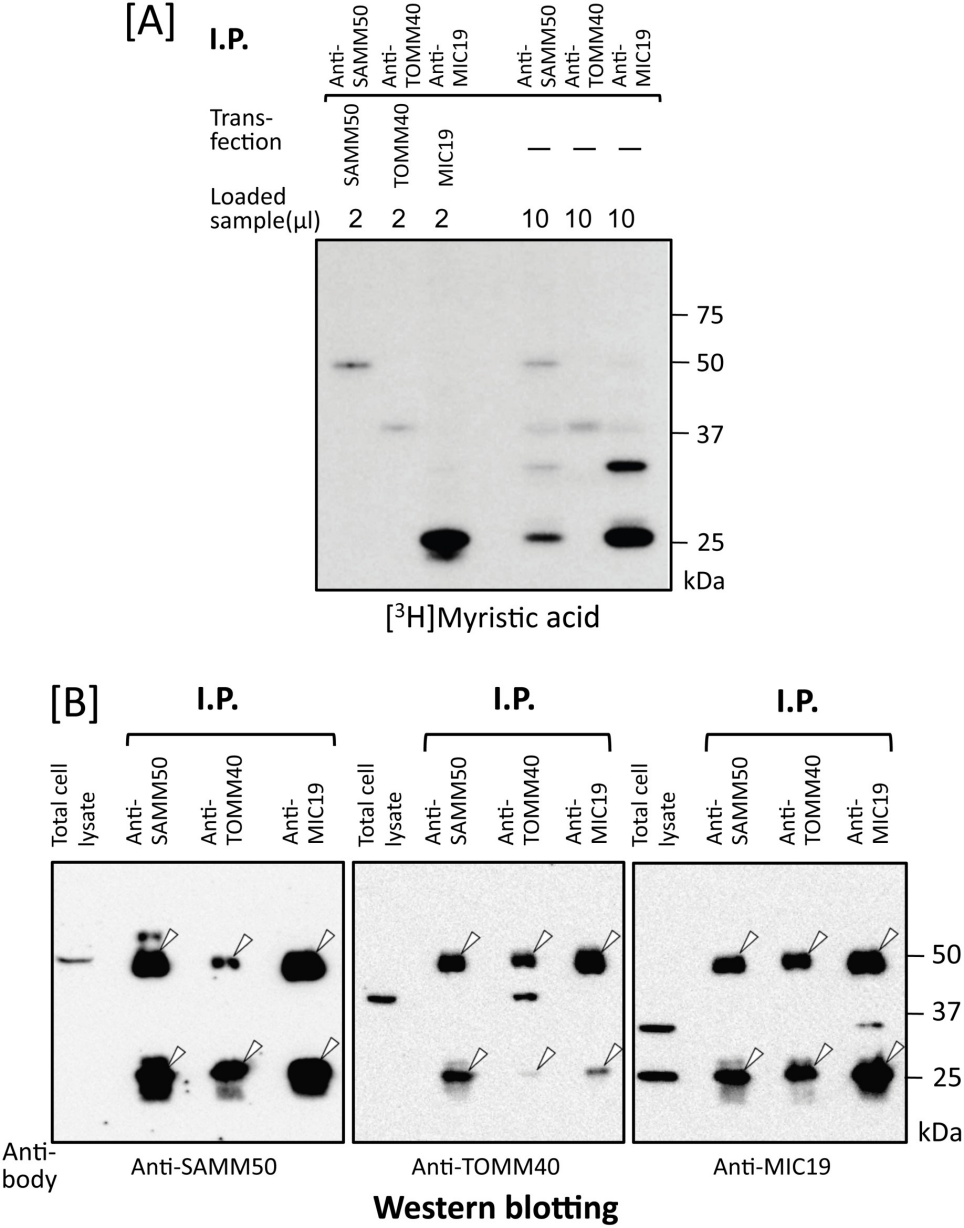
Identification of MIC19 as a major *N*-myristoylated binding partner of SAMM50. A. Metabolic labeling of *N*-myristoylated proteins expressed in COS-1 cells with [^3^H]myristic acid followed by immunoprecipitation with specific antibodies against SAMM50, TOMM40, and MIC19 was performed. The labeled proteins were separated by SDS-PAGE and then detected by fluorography (lanes 4–6). For this analysis, tag-free-SAMM50, -TOMM40, and -MIC19 exogenously expressed in COS-1 cells were used as controls (lanes 1–3). B. Western blotting analysis of immunoprecipitated samples used in A. Presence of endogenous SAMM50, TOMM40, and MIC19 in the immunoprecipitated samples used in A lanes 4–6 was determined by western blotting analysis. For this analysis, total cell lysates of COS-1 cells were used as control (lanes 1, 5, and 9). Arrowheads in B indicate the position of heavy- and light-chains of IgG used for immunoprecipitation.

As shown in Fig 4A, lane 2, several [^3^H]myristic acid-labeled protein bands with molecular masses smaller than that of SAMM50 (50 kDa) were detected in the immunoprecipitated sample obtained using the anti-SAMM50 antibody. No such extra [^3^H]myristic acid-labeled protein bands were observed in the same experiment performed with the anti-TOMM40 antibody (Fig 4A, lane 3). Surprisingly, the level of [^3^H]myristic acid incorporation into a 25 kDa protein was much higher than that into SAMM50. Because it was reported that MIC19, an *N*-myristoylated mitochondrial protein with a molecular mass of 25 kDa, specifically interacted with SAMM50 [22–24], we next studied whether the 25 kDa [^3^H]myristic acid-labeled protein band was MIC19 or not. When western blotting analysis of total cell lysates of COS-1 cells was performed, a specific protein band with a molecular mass of 25 kDa and a faint ~32 kDa protein band were detected using an anti-MIC19 antibody (Fig 4B, lane 5). In addition, the molecular mass of major endogenous MIC19 was exactly the same as that observed with tag-free MIC19 overexpressed in COS-1 cells (Fig 4B, lane 6), suggesting that the [^3^H]myristic acid-labeled protein band with a molecular mass of 25 kDa was endogenous *N*-myristoylated MIC19. When COS-1 cells were labeled with [^3^H]myristic acid and then immunoprecipitated with anti-MIC19 antibody, a specific protein band with a molecular mass of 25 kDa and a faint ~32 kDa protein band were detected by SDS-PAGE and fluorography, as shown in Fig 5A, lane 6. However, the ~32 kDa [^3^H]myristic acid-labeled protein band was not generated when a cDNA coding for tag-free MIC19 was transfected into COS-1 cells (Fig 5A, lane 3). These results suggested that the ~32 kDa [^3^H]myristic acid-labeled protein band was not a translation product of the cDNA coding for 25 kDa MIC19 but might be an isoform of MIC19. The fact that both the 25 kDa and ~32 kDa [^3^H]myristic acid-labeled protein bands were detected in the immunoprecipitated sample obtained using the anti-SAMM50 antibody indicated that both of these MIC19 isoforms were associated with SAMM50 (Fig 5A, lane 4). Thus, MIC19 and its ~32 kDa isoform were found to be major *N*-myristoylated binding partners of SAMM50.

### Identification and characterization of protein *N*-myristoylation occurring on human MIC25

In addition to MIC19, it was recently demonstrated that MIC25, a member of CHCH domain-containing protein family that has been predicted to be *N*-myristoylated, interacted with SAMM50 [25]. Therefore, we next studied whether MIC25 was contained in the immunoprecipitated sample obtained using the anti-SAMM50 antibody.

**Fig 6:**
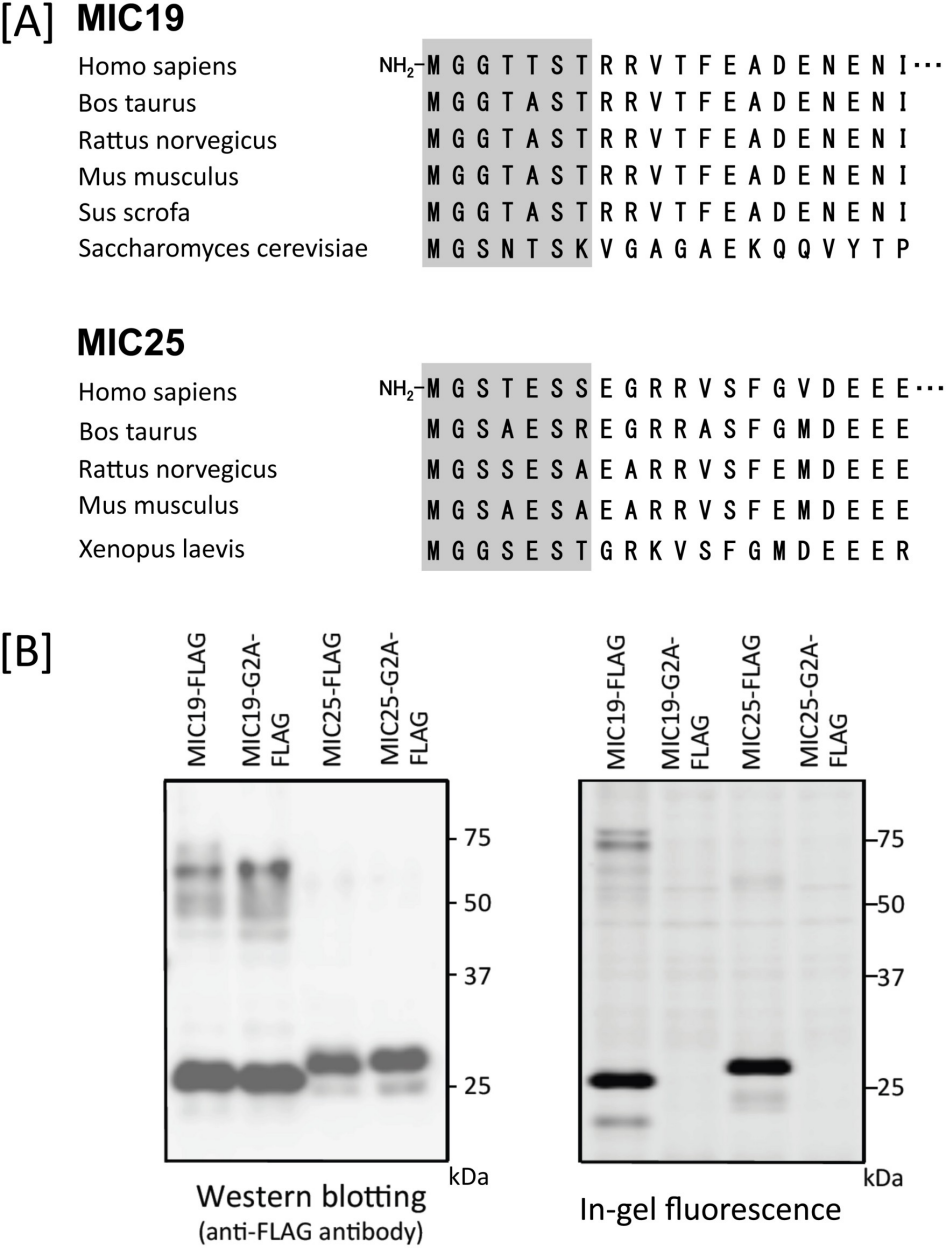
Detection of protein *N*-myristoylation occurring on human MIC19 and MIC25 by cellular metabolic labeling. A. Interspecies alignment of the N-terminal sequences of MIC19 and MIC25. *N*-myristoylation motifs are shown in grey in the N-terminal sequences. B. cDNAs encoding MIC19-FLAG, MIC25-FLAG, and their G2A mutants were transfected into COS-1 cells. The expression of proteins was evaluated by western blotting analysis using anti-FLAG antibodies. Protein *N*-myristoylation was evaluated by metabolic labeling with a myristic acid analog followed by click chemistry, as described in the Materials and methods.

Because protein *N*-myristoylation of MIC25 has not been verified experimentally, we first studied the protein *N*-myristoylation occurring on MIC25. For this analysis, MIC19 was used as a control *N*-myristoylated protein. As shown in Fig 6A, interspecies alignments of the N-terminal sequence of the proteins revealed that the N-terminal *N*-myristoylation motif of MIC25 was highly conserved among vertebrates, as was the case with MIC19. In addition, both *N*-myristoylation prediction programs (The MYR Predictor and Myristoylator) predicted MIC25 to be an *N*-myristoylated protein. To determine whether MIC25 is *N*-myristoylated, cellular metabolic labeling experiments were performed using cDNAs coding for wild-type and G2A mutant of C-terminally FLAG-tagged MIC25. As shown in Fig 6B, left panel, efficient expression of proteins with the predicted molecular mass (26 kDa plus 1 kDa FLAG) in COS-1 cells were observed with wild-type and G2A mutant of MIC25-FLAG, as was the case with those of MIC19-FLAG determined by western blotting analysis. Metabolic labeling experiments in COS-1 cells using bioorthogonal myristic acid analog (Alk-Myr) followed by detection with click chemistry revealed that efficient protein *N*-myristoylation was observed in wild-type MIC19-FLAG and MIC25-FLAG, but protein *N*-myristoylation was completely inhibited by replacing Gly2 with Ala, as shown in Fig 6B, right panel. These results clearly showed that MIC19 and MIC25 are efficiently *N*-myristoylated in transfected mammalian cells.

### Protein *N*-myristoylation of MIC19 but not of MIC25 is required for proper targeting to mitochondria

**Fig 7:**
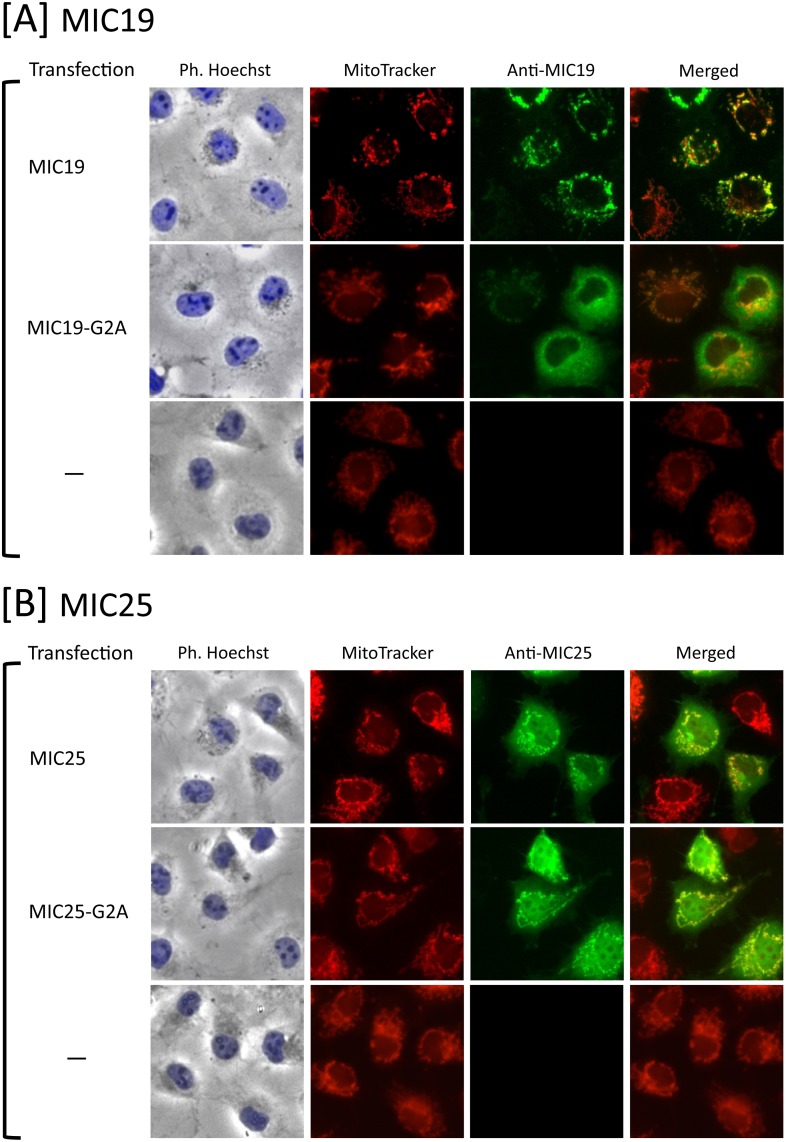
Protein *N*-myristoylation of MIC19 but not of MIC25 is required for proper targeting to mitochondria. A. Intracellular localization of MIC19 and MIC19-G2A was determined by immunofluorescence staining of COS-1 cells transfected with cDNAs encoding these two proteins using an anti-MIC19 antibody. Mitochondria were detected using MitoTracker Red. The same experiment was performed using non-transfected COS-1 cells as a control. B. Intracellular localization of MIC25 and MIC25-G2A was determined by immunofluorescence staining of COS-1 cells transfected with cDNAs encoding these two proteins using an anti-MIC25 antibody. Mitochondria were detected using MitoTracker Red. The same experiment was performed using non-transfected COS-1 cells as a control.

**Fig 8:**
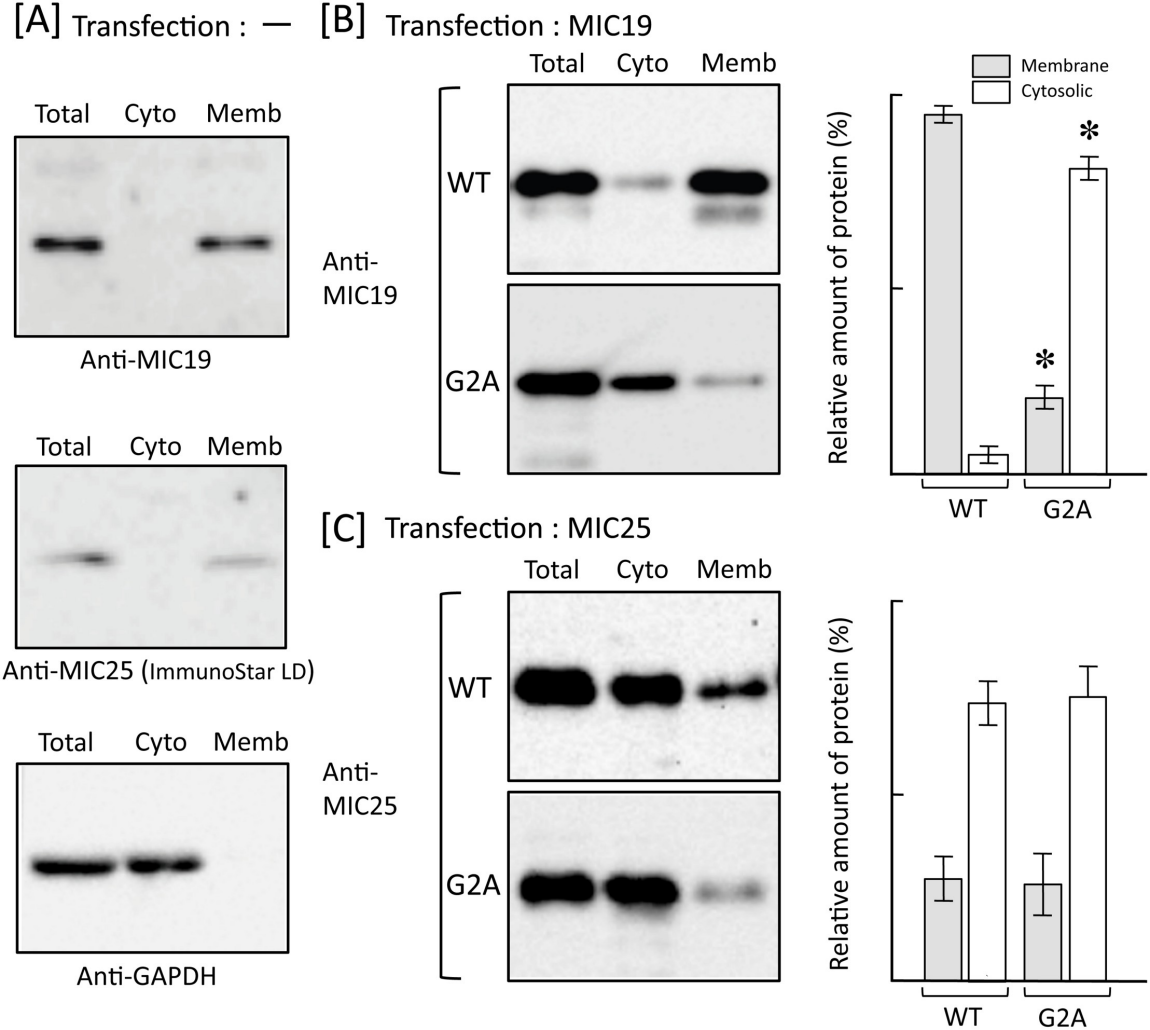
Protein *N*-myristoylation of MIC19 but not of MIC25 is required for binding of protein to the membrane. Total cell extracts of non-transfected COS-1 cells [A] and COS-1 cells transfected with cDNA encoding tag-free MIC19 [B] or MIC25 [C] were separated into cytosolic and membrane fractions by using membrane protein extraction kit (Trident). Presence of MIC19 and MIC25 in each fraction was determined by western blotting analysis using anti-MIC19 or anti-MIC25 antibody, respectively. For this analysis, anti-GAPDH antibody was used to detect GAPDH, a cytoslic marker protein [A]. For the analysis of COS-1 cells transfected with cDNAs coding for MIC19 [B] and MIC25 [C], relative amount of protein (%) reside in the cytosolic and total membrane fraction was calculated from densitometric analysis as described in materials and methods. Data are expressed as mean ± SD for three independent experiments. *P < 0.005 vs. wild-type.

To determine the physiological role of protein *N*-myristoylation occurring on human MIC25, the role of protein *N*-myristoylation in intracellular localization was investigated by immunofluorescence staining of COS-1 cells transfected with cDNA encoding tag-free MIC25. For this analysis, MIC19 in which protein *N*-myristoylation has been shown to play a critical role in mitochondrial targeting [23] was used as a control. As shown in Fig 7A and 7B, a striking difference in the role of protein *N*-myristoylation in mitochondrial targeting was observed between MIC19 and MIC25. Immunofluorescence staining with the anti-MIC19 antibody coupled with MitoTracker staining revealed that *N*-myristoylated MIC19 exclusively localized to mitochondria, whereas the non-myristoylated G2A mutant localized mainly to the cytoplasm. In contrast, in the case of MIC25, both the wild-type and G2A mutant were found to localize mainly to cytoplasm and only a small amount of protein was found in mitochondria. Thus, *N*-myristoylation of MIC25 did not play a critical role in the mitochondrial targeting of this protein. In these experiments, endogenous MIC19 and MIC25 in control non-transfected COS-1 cells were not detected under the same experimental condition used for the analysis of the transfected COS-1 cells as shown in the lower panels of Fig 7A and 7B. In order to further clarify the role of protein *N*-myristoylation in the intracellular localization of MIC19 and MIC25, the role of protein *N*-myristoylation in the membrane binding of these two proteins was investigated by subcellular fractionation analysis using membrane protein extraction kit as performed in Fig 3. As shown in Fig 8A, endogenous MIC19 and MIC25 expressed in control non-transfected COS-1 cells were detected exclusively in total membrane fraction, whereas GAPDH, a cytosolic marker protein, was detected exclusively in cytosolic fraction. In the case of detection of endogenous MIC25, the expression could not be detected by using conventional western blotting detection reagent (data not shown), and the MIC25 protein band was detected only when highly sensitive western blotting detection reagent (ImmunoStar LD) was used. These data indicated that the membrane proteins were successfully separated from the cytosolic proteins by using membrane protein extraction kit. As shown in Fig 8B and 8C, a striking difference in the role of protein *N*-myristoylation in membrane binding was observed between MIC19 and MIC25. Western blotting analysis using anti-MIC19 antibody revealed that most of the overexpressed *N*-myristoylated MIC19 localized to the membrane, whereas most of the non-myristoylated G2A mutant localized to the cytoplasm. In contrast, as for MIC25, many of the wild-type and G2A mutant were found to localize to the cytoplasm, and only a small portion of the protein was detected in the membrane. Thus, *N*-myristoylation of MIC19 but not of MIC25 plays critical role in the binding of protein to the membrane.

### Endogenous MIC25 expressed in mammalian cells is *N*-myristoylated

**Fig 9:**
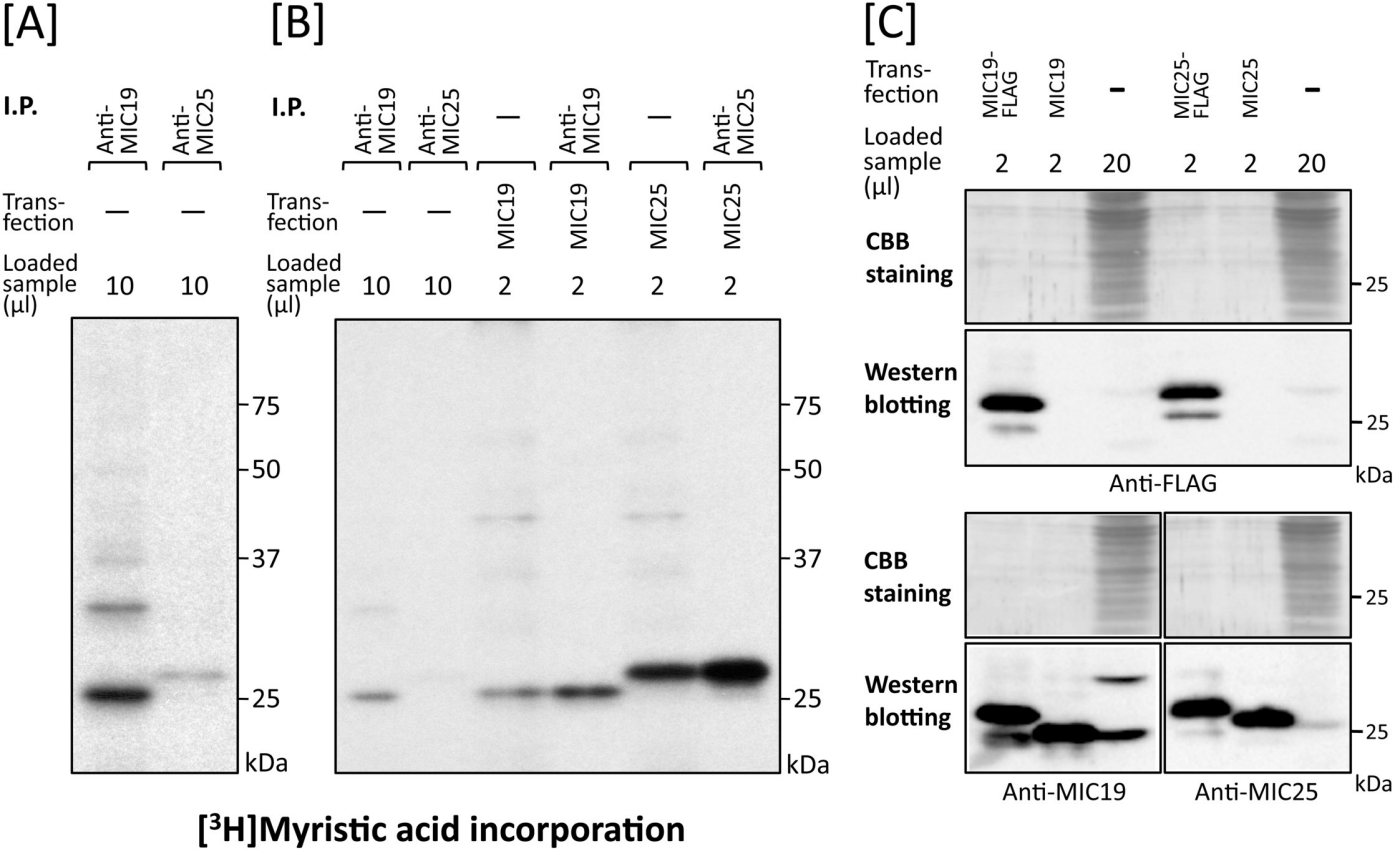
Expression and *N*-myristoylation of endogenous MIC19 and MIC25 in COS-1 cells. A. Metabolic labeling of endogenous proteins expressed in COS-1 cells with [^3^H]myristic acid followed by immunoprecipitation with specific antibodies against MIC19 and MIC25 was performed. The labeled proteins were separated by SDS-PAGE and then detected by fluorography. B. Comparison of the molecular size of endogenous *N*-myristoylated MIC19 and MIC25 immunoprecipitated from COS-1 cells with that of *N*-myristoylated tag-free MIC19 and MIC25 exogenously expressed in COS-1 cells. [^3^H]myristic acid labeling of COS-1 cells transfected with cDNA coding for tag-free MIC19 or MIC25 followed by immunoprecipitation with specific antibodies against these two proteins was performed. The immunoprecipitated samples and the samples obtained in A were separated by SDS-PAGE and then detected by fluorography. C. Comparison of the relative amounts of MIC19 and MIC25 expressed in COS-1 cells. The expression level of endogenous MIC25 in COS-1 cells was compared with that of MIC19 by western blotting analysis using MIC19-FLAG and MIC25-FLAG as standard proteins. For this analysis, total cell lysates derived from non-transfected COS-1 cells or COS-1 cells transfected with cDNAs coding for MIC19-FLAG, tag-free MIC19, MIC25-FLAG, or tag-free MIC25 were subjected to western blotting analysis using anti-FLAG, anti-MIC19, or anti-MIC25 antibodies.

**Fig 10:**
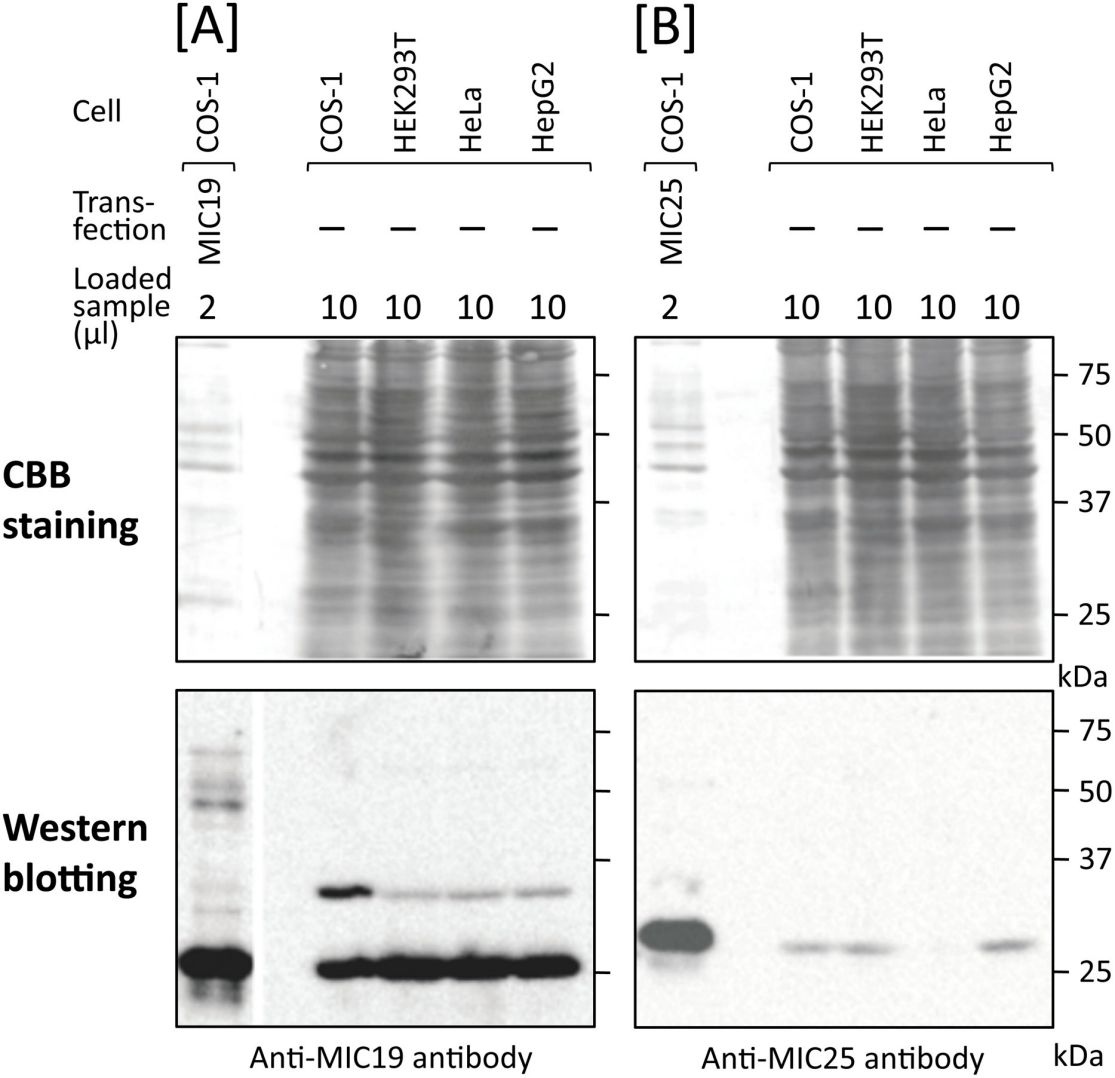
Comparison of the relative amounts of MIC19 and MIC25 expressed in mammalian cells. The expression levels of endogenous MIC25 in four mammalian cell lines (COS-1, HEK293T, HeLa, HepG2 cells) were compared with those of MIC19 by western blotting analysis using tag-free MIC19 and tag-free MIC25 expressed in COS-1 cells as standard proteins. For this analysis, total cell lysates derived from the four cell lines were subjected to western blotting analysis using anti-MIC19 or anti-MIC25 antibodies. A; Expression of MIC19, B; Expression of MIC25, Upper panels; Coomassie-brilliant blue (CBB) staining, Lower panels; Western blotting analysis.

To determine whether endogenous MIC25 expressed in mammalian cells is *N*-myristoylated, COS-1 cells were labeled with [^3^H]myristic acid and then immunoprecipitation was performed using the anti-MIC25 antibody. As a result, a faint [^3^H]myristic acid-labeled protein band with a molecular mass of 26 kDa was detected by SDS-PAGE and fluorography, as shown in Fig 9A, lane 2. The molecular mass of the [^3^H]myristic acid-labeled endogenous MIC25 was exactly the same as that observed with [^3^H]myristic acid-labeled tag-free MIC25 overexpressed in COS-1 cells, suggesting that the [^3^H]myristic acid-labeled 26 kDa protein was endogenous *N*-myristoylated MIC25 (Fig 9B, lanes 2, 5 and 6). Because the strength of the [^3^H]myristic acid-labeled protein band of MIC25 was so weak compared with that of MIC19, it is probable that the level of protein expression of endogenous MIC25 was very low. To confirm this, the level of expression of endogenous MIC25 was compared with that of MIC19 by western blotting analysis using MIC19-FLAG, tag-free MIC19, MIC25-FLAG, and tag-free MIC25 expressed in COS-1 cells as standard proteins. As shown in Fig 9C, upper panels, lanes 1 and 4, similar amounts of MIC19-FLAG and MIC25-FLAG were expressed in transfected COS-1 cells. Whereas tag-free MIC19 and MIC25 were expressed at similar protein expression levels compared with FLAG-tagged MIC19 and MIC25, as determined using anti-MIC19 or anti-MIC25 antibodies, respectively (Fig 9C, lower panels, lanes 1, 2, 4, and 5). In the case of endogenous MIC19 and MIC25, only low levels of protein expression of MIC25 were observed in total cell lysates of COS-1 cells compared with that of MIC19 (Fig 9C, lower panels, lanes 3 and 6). We next compared the expression levels of endogenous MIC19 and MIC25 in 4 different mammalian cell lines (COS-1, HEK293T, HeLa, and HepG2). As a result, a significant difference in protein expression was observed between MIC19 and MIC25, as shown in Fig 10. The 25 kDa MIC19 protein was expressed efficiently in all four cell lines. As for the ~32 kDa MIC19 isoform, relatively low levels of expression were observed in three human cell lines (HEK293T, HeLa, and HepG2) compared with monkey COS-1 cells (Fig 10, left panels). Concerning MIC25, a difference in protein expression levels was observed among the 4 cell lines; low expression levels were detected equally in COS-1, HEK293T, and HepG2 cells, whereas only very low expression levels were detected in HeLa cells, as shown in Fig 10, right panels.

### Protein *N*-myristoylation of MIC19 is required for association of MIC19 with SAMM50

**Fig 11:**
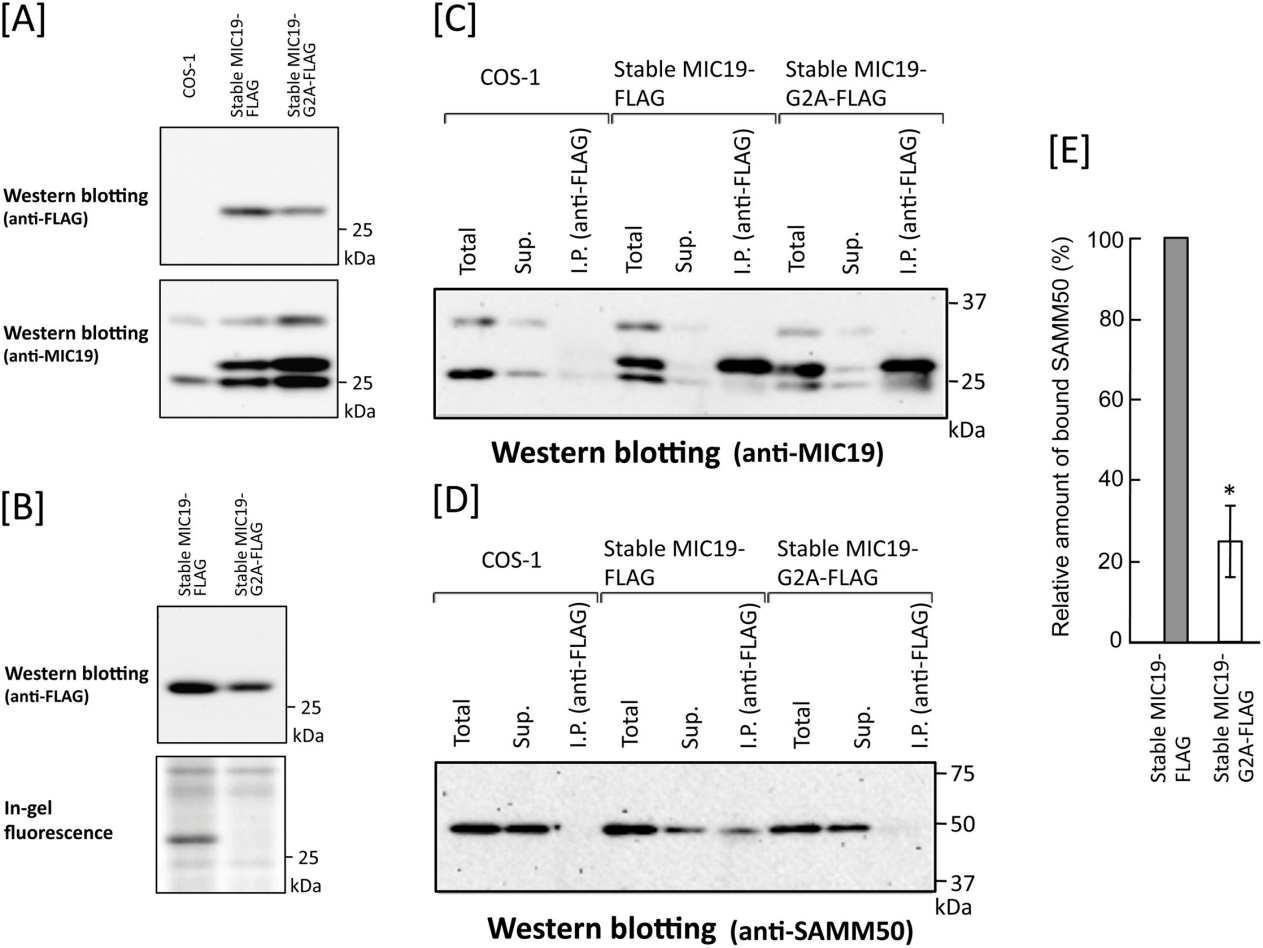
Protein *N*-myristoylation of MIC19 is required for the association of MIC19 with SAMM50. Association of MIC19-FLAG stably expressed in COS-1 cells with endogenously expressed SAMM50 was studied by immunoprecipitation analysis. A. Expression of MIC19-FLAG and MIC19-G2A-FLAG in respective stable transformants. Upper panel; Western blotting analysis using an anti-FLAG antibody, Lower panel; Western blotting analysis using an anti-MIC19 antibody. B. Detection of protein *N*-myristoylation of MIC19-FLAG expressed in stable transformants of MIC19-FLAG. Upper panel; Western blotting analysis using an anti-FLAG antibody. Lower panel; Detection of protein *N*-myristoylation by metabolic labeling with myristic acid analog followed by detection with click chemistry. C. Western blotting analysis of MIC19-FLAG or MIC19-G2A-FLAG immunoprecipitated from stable transformants of MIC19-FLAG and MIC19-G2A-FLAG using an anti-MIC19 antibody. D. Analysis of the binding of MIC19-FLAG or MIC19-G2A-FLAG to endogenous SAMM50 by western blotting analysis of immunoprecipitated samples of stable transformants of MIC19-FLAG and MIC19-G2A-FLAG using an anti-SAMM50 antibody. The experiments were performed in triplicate. A typical pattern of western blotting is presented. E. Quantitative analysis of MIC19-FLAG or MIC19-G2A-FLAG bound to SAMM50 was performed using the results obtained in D. Data are expressed as mean ± SD for three independent experiments. *P < 0.01 vs. wild-type.

We next studied the role of protein *N*-myristoylation on the protein-protein interaction between SAMM50 and MIC19. As shown in Fig 5A, lane 1, association of SAMM50 with MIC19 was not observed in COS-1 cells transiently transfected with SAMM50 cDNA. Therefore, we tried to establish transformants stably expressing wild-type and G2A mutant of C-terminally FLAG-tagged SAMM50 or MIC19. As a result, stable transformants of MIC19-FLAG were successfully established, as shown in Fig 11A and 11B. However, as for wild-type and G2A mutant of SAMM50-FLAG, we tried more than five times but could not obtain COS-1 cells stably expressing these proteins. When the association of stably expressed MIC19-FLAG or MIC19-G2A-FLAG with endogenously expressed SAMM50 in the two stable transformants was studied by immunoprecipitation using anti-FLAG antibody, a striking difference was observed between two transformants, as shown in Fig 11C and 11D. In both transformants, FLAG-tagged MIC19 protein was efficiently immunoprecipitated with an anti-FLAG antibody (Fig 11C, lanes 6 and 9). However, endogenous SAMM50 was detected only in the immunoprecipitated sample of the stable transformant expressing wild-type MIC19-FLAG (Fig 11D, lane 6). Quantitative analysis of the amount of SAMM50 bound to MIC19-FLAG or MIC19-G2A-FLAG confirmed the efficient binding of SAMM50 to MIC19-FLAG but not to MIC19-G2A-FLAG, as shown in Fig 11E. These results clearly indicated that protein *N*-myristoylation of MIC19 is required for the association of MIC19 with SAMM50.

## Discussion

Protein *N*-myristoylation has been recognized as a protein modification that occurs mainly on cytoplasmic proteins. In eukaryotic cellular proteins, only very few integral membrane proteins have been demonstrated to be *N*-myristoylated. In this study, we first characterized protein *N*-myristoylation occurring on two human mitochondrial membrane proteins, SAMM50 and TOMM40. SAMM50 and TOMM40 are β-barrel proteins that reside within the outer membrane of mitochondria [19–21, 39]. SAMM50 is a central component of the SAM complex necessary for the assembly of β-barrel proteins in the mitochondrial outer membrane [19, 21]. TOMM40 is the protein conducting channel of the translocase of the outer mitochondrial membrane (TOM) acts as a general import pore for most mitochondrial precursor proteins [20, 27]. As for mitochondrial localization of TOMM40 and SAMM50, specific mitochondrial targeting signal has not been determined. Both of these proteins are β-barrel proteins and contain a β-signal that is required for β-barrel precursor recognition by the SAM complex [38]. However, it was reported that the β-signal is not required for mitochondrial targeting of yeast β-barrel proteins [38]. Thus, so far, the mechanisms for specific mitochondrial targeting of TOMM40 and SAMM50 remains to be elucidated.

Metabolic labeling experiments using transfected mammalian cells performed in this study revealed that both SAMM50 and TOMM40 are *N*-myristoylated (Fig 1C). It should be noted that N-terminal *N*-myristoylation motifs were observed exclusively in vertebrates (Fig 1A). Therefore, SAM50 and TOM40 in yeast are not subjected to protein *N*-myristoylation. Analysis of intracellular localization in wild-type and a non-myristoylated G2A mutant of human proteins expressed in mammalian cells by immunofluorescence microscopic analysis revealed that protein *N*-myristoylation plays critical role in the mitochondrial targeting of SAMM50, but not that of TOMM40 (Fig 2). In accordance with these observations, subcellular fractionation analysis revealed that protein *N*-myristoylation of SAMM50 but not of TOMM40 plays positive role in the binding of protein to the membrane (Fig 3). β-barrel proteins do not enter the outer mitochondrial membrane from the cytosolic side but rather are transferred via the TOM complex into the intermembrane space and are subsequently directed to the outer membrane [21, 39]. Similar to other mitochondrial precursors, β-barrel proteins are first recognized by TOM receptors [40, 41] before they are imported across the outer membrane with the help of the TOM complex [41]. Therefore, it is probable that protein *N*-myristoylation of SAMM50 positively affected the interaction of SAMM50 with TOM receptors. In fact, non-myristoylated G2A mutant of SAMM50 did not localize to a membranous compartment but to the cytoplasm (Figs 2A and 3B). In contrast to SAMM50, protein *N*-myristoylation of TOMM40 did not affect mitochondrial targeting and membrane binding of TOMM40. The mechanism for the difference in the role of protein *N*-myristoylation on the mitochondrial targeting and membrane binding of these two proteins is not clear.

When protein *N*-myristoylation occurring on endogenous proteins expressed in COS-1 cells was analyzed by immunoprecipitation using specific antibodies, a striking difference was observed in the immunoprecipitated proteins obtained using anti-SAMM50 and anti-TOMM40 antibodies (Fig 4A). In the case of TOMM40, [^3^H]myristic acid-labeled 40 kDa single protein band was detected in the fluorogram. Western blotting analysis further confirmed the presence of TOMM40 in the immunoprecipitated sample obtained using the anti-TOMM40 antibody (Fig 5B, lane 7). As for SAMM50, in addition to a [^3^H]myristic acid-labeled 50 kDa SAMM50 band, multiple protein bands were detected in the fluorogram. Comparison of the molecular mass of these protein bands with those of the tag-free MIC19, and MIC25 protein expressed in COS-1 cells, or endogenous [^3^H]myristic acid-labeled proteins in COS-1 cells immunoprecipitated using anti-MIC19 or anti-MIC25 antibodies clearly indicated that major *N*-myristoylated binding partner of SAMM50 is MIC19 and its ~32 kDa isoform, but not MIC25 (Figs 4, 5 and 9). So far, the isoform of MIC19 has not been reported. Thus, this is the first report indicating the presence of a MIC19 isoform with higher molecular mass. Of note, much higher incorporation of [^3^H]myristic acid was detected in the 25 kDa MIC19 protein band as compared with the 50 kDa SAMM50 band. Because protein *N*-myristoylation occurs at a single N-terminal Gly residue in each *N*-myristoylated protein, it was suggested that several molecules of *N*-myristoylated MIC19 bound to each SAMM50 molecule.

Coiled-coil-helix-coiled-coil-helix domain (CHCHD)-containing proteins are nucleus-encoded small mitochondrial proteins with important functions [42, 43]. Nine members have been identified in this protein family. All CHCHD proteins have at least one functional CHCHD, which is stabilized by two pairs of disulfide bonds between two helices. CHCHD proteins have various important pathophysiological roles in mitochondria and other key cellular processes. MIC19 and MIC25 are part of the CHCHD protein family. Phylogenic analysis indicated that human MIC19 and MIC25 resulted from a gene duplication at the root of the vertebrates [44]. Thus, they are both orthologous to MIC19 in yeast and likely the result of the whole genome duplication at the root of the vertebrates, a pattern that has been observed for mitochondrial proteins [45]. Concerning mitochondrial localization of MIC19 and MIC25, neither canonical cleavable N-terminal mitochondrial targeting signal (presequence) nor other specific mitochondrial targeting signal has been determined. However, as for MIC19, it was reported that both protein *N*-myrstoylation and CHCHD located near the C-terminus were essential for proper mitochondrial targeting of this protein [23]. In this case, however, the mechanisms by which protein *N*-myristoylation and CHCHD direct the mitochondrial targeting have not been elucidated.

MIC19 is an *N*-myristoylated inner membrane-bound protein. MIC19 interacts with the peripheral surface of the mitochondrial contact site and cristae organizing system (MICOS) complex, facing the intermembrane space, where its presence is necessary for cristae formation and communication with the outer mitochondrial membrane [23, 24, 26]. As for MIC25, it was predicted to be *N*-myristoylated, however, direct biochemical evidence for the protein *N*-myristoylation has not been demonstrated. As shown in Fig 6A, the *N*-myristoylation motif of MIC25 is highly conserved among vertebrates. In the present study, we showed that exogenously and endogenously expressed MIC25 is efficiently *N*-myristoylated. Interestingly, however, most of the *N*-myristoylated MIC25 overexpressed in COS-1 cells was detected in the cytoplasm but not in mitochondria (Figs 7 and 8). Thus, protein *N*-myristoylation does not play critical role in the mitochondrial targeting or membrane binding of MIC25. The mechanism for the difference in the role of protein *N*-myristoylation on the mitochondrial targeting and membrane binding of MIC19 and MIC25 is not clear.

The very large MICOS complex is located in the inner membrane and connects with proteins in the outer membrane. To date, eight MICOS subunits in mammals (MIC10, MIC13, MIC14, MIC19, MIC23, MIC25, MIC27, and MIC60) have been identified [26, 46, 47]. Most of the MICOS subunits are integral membrane proteins in the mitochondrial inner membrane, however, MIC19 and MIC25 are peripheral membrane proteins. MIC19 and MIC25 are both CHCHD proteins and are characterized by the presence of CX_9_C motifs with two cysteines that stabilize coiled-coil-helix domains by forming disulphide bonds. These physicochemical properties are associated with scaffold functions, reflecting the structural and protein-protein interaction roles of these proteins. MIC19 is a 26 kDa protein associated with the inner membrane, interacting with MIC60 through the coiled coil helix motif in the C-terminal domain, and with SAMM50 through the N-terminal domain [23]. Therefore, MIC19 bridges MIC60 and SAMM50, contributing to the formation of contact sites. This structure is called the MIB complex, and it is crucial for the maintenance of cristae and the assembly of respiratory chain complexes [48, 49]. The fact that MIC19 interacts with SAMM50 through the *N*-myristoylated N-terminal domain and that SAMM50 exposes an N-terminal polypeptide transport-associated domain into the intermembrane space suggested that N-terminal *N*-myristoylation of both MIC19 and SAMM50 might be involved in the specific interaction of MIC19 and SAMM50. Concerning the role of protein *N*-myristoylation of MIC19 in the interaction of MIC19 and SAMM50, it was previously reported that MIC19 transiently expressed in HEK293 cells bound to endogenous SAMM50 in an *N*-myristoylation dependent manner [24]. On the contrary, in the present study it was shown that MIC19 transiently expressed in COS-1 cells could not bind to endogenous SAMM50, as shown in Fig 5A, lane 3. The reason for the discrepancy between these observations was not clear. However, in this study, MIC19-FLAG stably expressed in COS-1 cells with an expression level similar to that of the endogenous MIC19 was found to bind to endogenous SAMM50 in an *N*-myristoylation-dependent manner (Fig 11C–11E), suggesting that the stoichiometry of the expressed proteins in the cells might affect the interaction of MIC19 with SAMM50. In order to determine the intracellular localization of stably expressed MIC19-FLAG and MIC19-G2A-FLAG, we performed immunofluorescence analysis using anti-FLAG antibody. As a result, different from the experimental results obtained with transiently expressed MIC19 and MIC19-G2A (Fig 7A), both of stably expressed MIC19-FLAG and MIC19-G2A-FLAG were found to localize specifically to mitochondria as shown in S1 Fig. Thus, it is probable that, in this experimental system, the lack of *N*-myristoylation did not affect the mitochondrial localization but affected the protein-protein interaction occurring in the mitochondria. Thus, it was confirmed that protein *N*-myristoylation of MIC19 plays a critical role in the MIC19-SAMM50 interaction. It is quite probable that *N*-myristoylation of SAMM50 also plays an important role in these interactions. Unfortunately, a stable transformant of SAMM50 has not been established even after multiple attempts. Thus, the role of protein *N*-myristoylation of SAMM50 on the SAMM50-MIC19 interaction remains to be elucidated.

**Fig 12:**
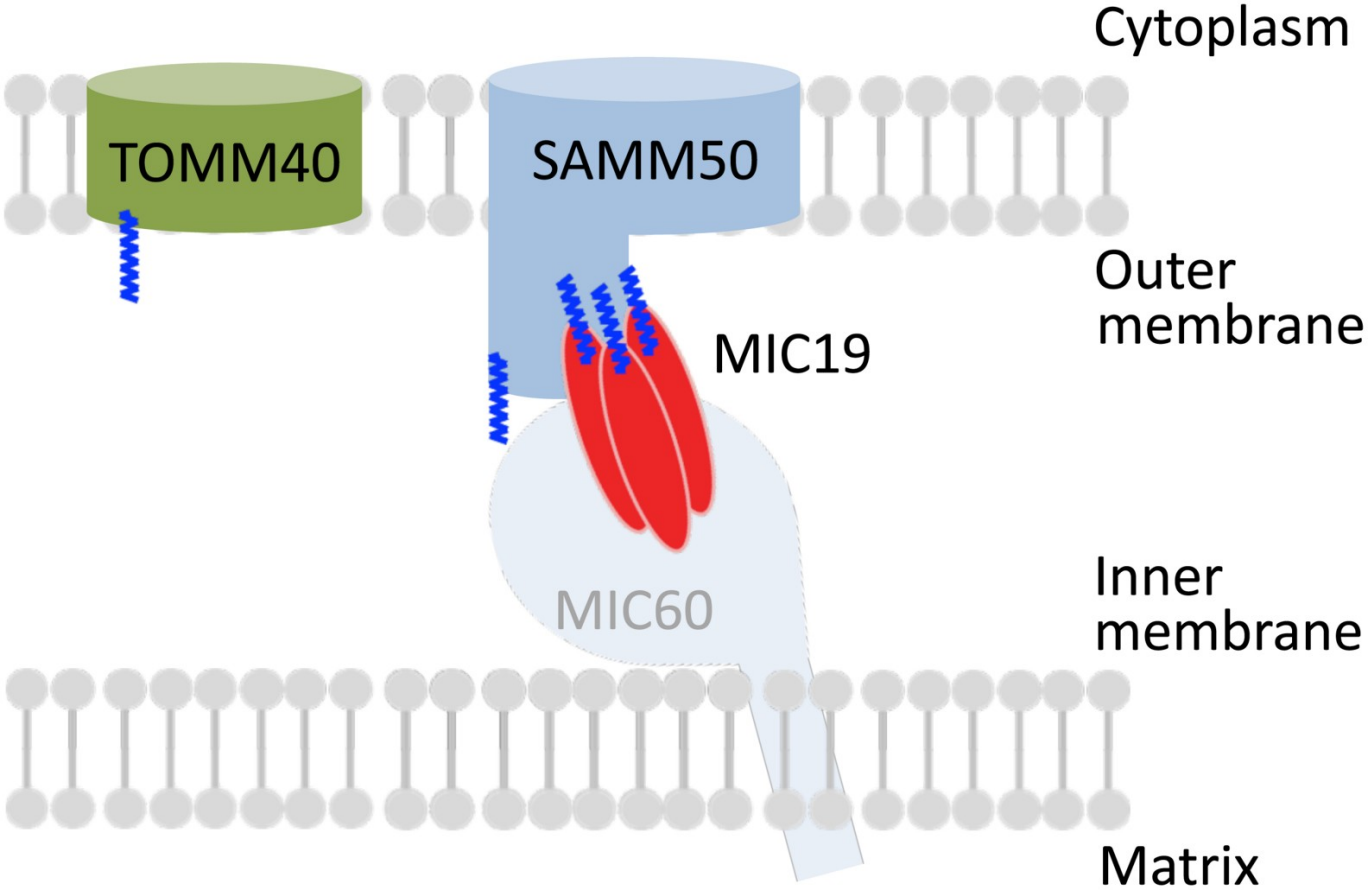
Schematic representation of the intracellular localization and protein-protein interactions of SAMM50, TOMM40, and MIC19. SAMM50, TOMM40, MIC19, and MIC25 were cotranslationally *N*-myristoylated in the cytoplasm. These proteins are transported into the intermembrane space of mitochondria through the TOM complex. For SAMM50 and MIC19, protein *N*-myristoylation is required for mitochondrial targeting. An oligomeric form of *N*-myristoylated MIC19 seems to associate with *N*-myristoylated SAMM50. Protein *N*-myristoylation of MIC19 is required for the binding of MIC19 to SAMM50.

MIC25 is a 26 kDa protein also associated with the mitochondrial inner membrane. It has been poorly characterized thus far, but a recent immunoprecipitation study revealed the physical interaction of MIC25 with SAMM50 and MIC60 to sustain cristae structure [25, 50]. However, another study suggested that the most important subunits of the MIB complex in human mitochondria are Mic60, Mic19, and SAMM50, and that MIC25 is a peripheral subunit of the MICOS complex because its depletion did not affect cristae morphology [51]. In the present study, immunoprecipitation experiments using specific antibodies revealed that MIC19, but not MIC25, was a major *N*-myristoylated binding partner of SAMM50. It was also revealed that 25 kDa MIC19 and its ~32 kDa isoform were ubiquitously expressed in mammalian cells. However, the expression of MIC25 differed depending on the cell-type, and only a very low level of expression was detected in human HeLa cells. In fact, it was reported that MIC25 does not play a critical role in the formation and stability of cristae in HeLa cells [51]. Thus, although MIC19 was established as a crucial component of MICOS and MIB, MIC25 seems to fulfill a more peripheral function, as reported previously [42, 51]. Schematic representation of the intracellular localization and protein-protein interactions of SAMM50, TOMM40, and MIC19 is shown in Fig 12.

Protein *N*-myristoylation has been recognized as a protein modification that occurs mainly on cytoplasmic proteins, and the functions of these *N*-myristoylated proteins are often regulated by reversible membrane binding to the plasma membrane mediated by protein *N*-myristoylation as observed in the case of src family tyrosine kinases and G protein α subunits [1, 4–6]. In the present study, we showed that four mitochondrial proteins, two integral outer membrane proteins (SAMM50, TOMM40) and two mitochondrial intermembrane space proteins (MIC19, MIC25), were *N*-myristoylated. In addition, protein *N*-myristoylation was found to play a critical role in the mitochondrial targeting of two MIB components (SAMM50 and MIC19), and the protein-protein interaction between MIC19 and SAMM50 (Fig 11). Thus, the role of protein *N*-myristoylation in the intracellular targeting and physiological function of *N*-myristoylated proteins seems to be much more diverse than reported previously.

## Supporting information

S1 Fig Intracellular localization of MIC19-FLAG and MIC19-G2A- FLAG stably expressed in COS-1 cells. To determine the intracellular localization of stably expressed MIC19-FLAG and MIC19-G2A-FLAG, immunofluorescence analysis was performed using anti-FLAG antibody. As a result, different from the experimental results obtained with transiently expressed MIC19 and MIC19-G2A (Fig 7A), both of stably expressed MIC19-FLAG and MIC19-G2A-FLAG were found to localize specifically to mitochondria.
(TIF)

S1 Table Nucleotide sequences of oligonucleotides used in this study. (DOCX)

S2 Table The strategies for construction of pTD1 or pcDNA3 plasmids including cDNA clones. (DOCX)
