# Supplementary figures and images for: Identification and characterization of protein N-myristoylation occurring on four human mitochondrial proteins, SAMM50, TOMM40, MIC19, and MIC25
Source: PLoS One. 2018 Nov 14;13(11):e0206355. doi: 10.1371/journal.pone.0206355 (PMC6235283; doi:10.1371/journal.pone.0206355)

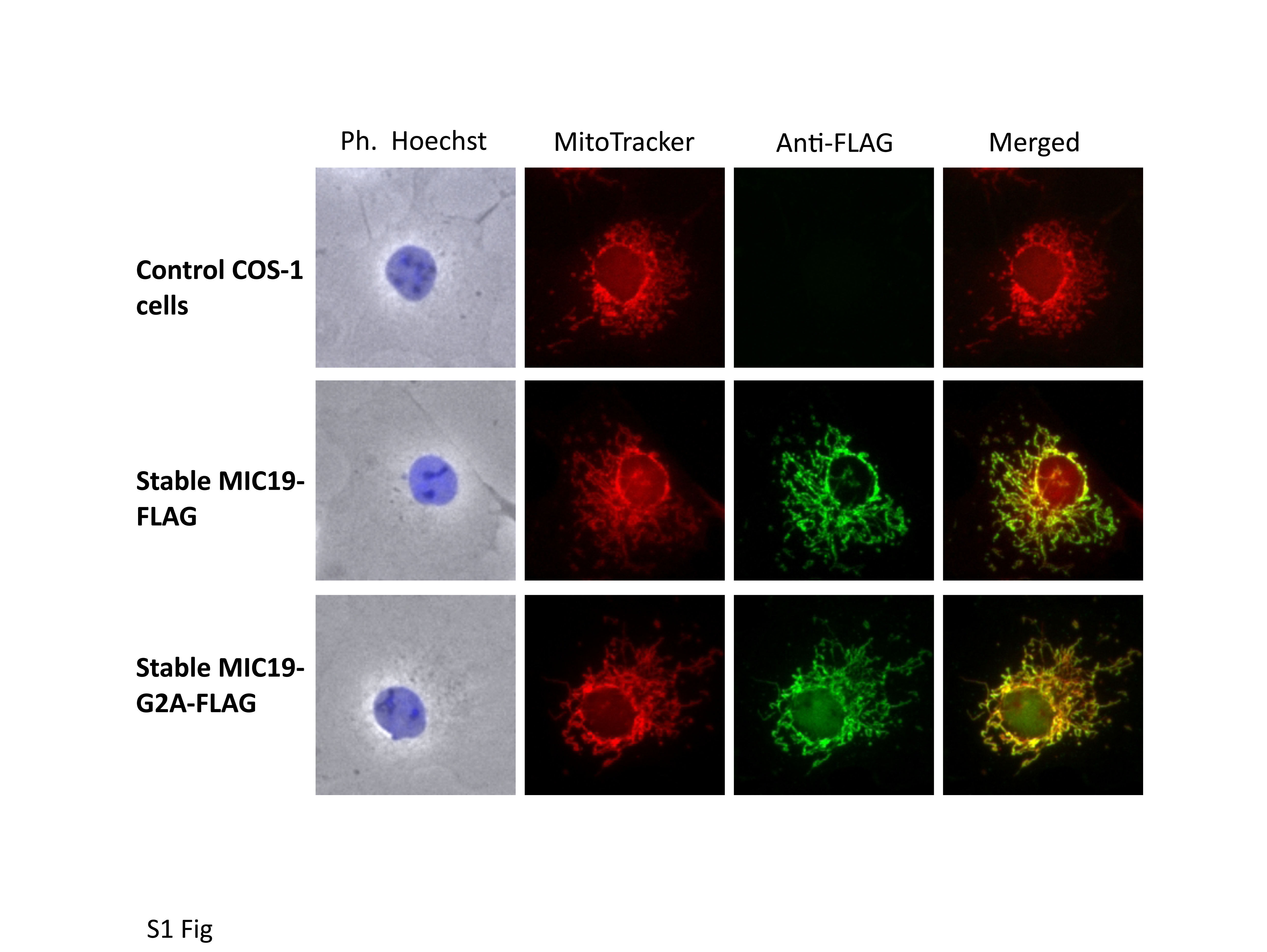

Supplement: S1 Fig — To determine the intracellular localization of stably expressed MIC19-FLAG and MIC19-G2A-FLAG, immunofluorescence analysis was performed using anti-FLAG antibody. As a result, different from the experimental results obtained with transiently expressed MIC19 and MIC19-G2A (Fig 7A), both of stably expressed MIC19-FLAG and MIC19-G2A-FLAG were found to localize specifically to mitochondria. (TIF) [file pone.0206355.s001.tif]
