# Supplementary material for: Identification and characterization of protein N-myristoylation occurring on four human mitochondrial proteins, SAMM50, TOMM40, MIC19, and MIC25
Source: PLoS One. 2018 Nov 14;13(11):e0206355. doi: 10.1371/journal.pone.0206355 (PMC6235283; doi:10.1371/journal.pone.0206355)
Supplement: S1 Table — (DOCX) [file pone.0206355.s002.docx]

S1 Table

The nucleotide sequences of oligonucleotides used in this study

| Sequence | |
| --- | --- |
| Primer-N1 | 5'-gcgcgatatcatggggaacgtgttg-3' |
| Primer-N2 | gcgcgatatcatggcgaacgtgttggctgcc |
| Primer-C1 | atatggatccgccgatggtgaggcc |
| Primer-N3 | atatggatccatggggactgtgcac |
| Primer-N4 | atatggatccatggccactgtgcacgcccgg |
| Primer-C2 | gcgatctagacaggaaccttat |
| Primer-C3 | gcgcctcagattacaggaaccttatccc |
| Primer-N5 | gcgcgaattcatggggaacgtgttg |
| Primer-N6 | gcgcgaattcatggcgaacgtgttggctgcc |
| Primer-C4 | gcgcgatatcgccgatggtgaggcc |
| Primer-C5 | atatgatatcctagccgatggtgaggcc |
| Primer-N7 | atatggatccatgggtgggaccacc |
| Primer-N8 | atatggatccatggctgggaccaccagcacc |
| Primer-C6 | gcgcgatatctcctcccttctcaag |
| Primer-C7 | gcgcgatatcttatcctcccttctcaag |
| Primer-N9 | gcatgaattcatggggagcacggag |
| Primer-N10 | gcatgaattcatggcgagcacggagagcagc |
| Primer-C8 | gcgcgatatcttacttgtcatcgtc |
| Primer-C9 | ctatgatatctcagcccttgtgggcggc |
