# Supplementary material for: Identification and characterization of protein N-myristoylation occurring on four human mitochondrial proteins, SAMM50, TOMM40, MIC19, and MIC25
Source: PLoS One. 2018 Nov 14;13(11):e0206355. doi: 10.1371/journal.pone.0206355 (PMC6235283; doi:10.1371/journal.pone.0206355)
Supplement: S2 Table — (DOCX) [file pone.0206355.s003.docx]

S2 Table

| Name of plasmid | Strategy for plasmid construction |
| --- | --- |
| pTD1-TOMM40-FLAG | product of a PCR with ORH08030 (Promega) as template and primers Primer-N1 and Primer-C1 was EcoRV/BamHI cloned into pTD1-FLAG |
| pTD1-TOMM40-G2A-FLAG | product of a PCR with ORH08030 (Promega) as template and primers Primer-N2 and Primer-C1 was EcoRV/BamHI cloned into pTD1-FLAG |
| pcDNA3-SAMM50-FLAG | product of a PCR with pF1KB8447(Promega) as template and primers Primer-N3 and Primer-C2 was BamHI/XbaI cloned into pcDNA3-FLAG |
| pcDNA3-SAMM50-G2A-FLAG | product of a PCR with pF1KB8447 (Promega) as template and primers Primer-N4 and Primer-C2 was BamHI/XbaI cloned into pcDNA3-FLAG |
| pcDNA3-SAMM50-Tag free | product of a PCR with pF1KB8447 (Promega) as template and primers Primer-N3 and Primer-C3 was BamHI/XhoI cloned into pcDNA3 |
| pcDNA3-TOMM40-FLAG | product of a PCR with ORH08030 (Promega) as template and primers Primer-N5 and Primer-C4 was EcoRI/EcoRV cloned into pcDNA3-FLAG |
| pcDNA3-TOMM40-G2A-FLAG | product of a PCR with ORH08030 (Promega) as template and primers Primer-N6 and Primer-C4 was EcoRI/EcoRV cloned into pcDNA3-FLAG |
| pcDNA3-TOMM40-Tag free | product of a PCR with ORH08030 (Promega) as template and primers Primer-N5 and Primer-C5 was EcoRI/EcoRV cloned into pcDNA3 |
| pcDNA3-MIC19-Flag | product of a PCR with pF1KB8182 (Promega) as template and primers Primer-N7 and Primer-C6 was EcoRI/EcoRV cloned into pcDNA3-FLAG |
| pcDNA3-MIC19-G2A-FLAG | product of a PCR with pF1KB8182 (Promega) as template and primers Primer-N8 and Primer-C6 was EcoRI/EcoRV cloned into pcDNA3-FLAG |
| pcDNA3-MIC19-Tag free | product of a PCR with pF1KB8182 (Promega) as template and primers Primer-N7 and Primer-C7 was EcoRI/EcoRV cloned into pcDNA3 |
| pcDNA3-MIC25-Flag | product of a PCR with IRAL018F23 (RikenBRC) as template and primers Primer-N9 and Primer-C8 was EcoRI/EcoRV cloned into pcDNA3-FLAG |
| pcDNA3-MIC25-G2A-FLAG | product of a PCR with IRAL018F23 (RikenBRC) as template and primers Primer-N10 and Primer-C8 was EcoRI/EcoRV cloned into pcDNA3-FLAG |
| pcDNA3-MIC25-Tag free | product of a PCR with IRAL018F23 (RikenBRC) as template and primers Primer-N9 and Primer-C9 was EcoRI/EcoRV cloned into pcDNA3 |
